# Cell size homeostasis is tightly controlled throughout the cell cycle

**DOI:** 10.1371/journal.pbio.3002453

**Published:** 2024-01-05

**Authors:** Xili Liu, Jiawei Yan, Marc W. Kirschner

**Affiliations:** 1 Department of Systems Biology, Harvard Medical School, Boston, Massachusetts, United States of America; 2 Department of Chemistry, Stanford University, Stanford, California, United States of America; The Institute of Cancer Research, UNITED KINGDOM

## Abstract

To achieve a stable size distribution over multiple generations, proliferating cells require a means of counteracting stochastic noise in the rate of growth, the time spent in various phases of the cell cycle, and the imprecision in the placement of the plane of cell division. In the most widely accepted model, cell size is thought to be regulated at the G1/S transition, such that cells smaller than a critical size pause at the end of G1 phase until they have accumulated mass to a predetermined size threshold, at which point the cells proceed through the rest of the cell cycle. However, a model, based solely on a specific size checkpoint at G1/S, cannot readily explain why cells with deficient G1/S control mechanisms are still able to maintain a very stable cell size distribution. Furthermore, such a model would not easily account for stochastic variation in cell size during the subsequent phases of the cell cycle, which cannot be anticipated at G1/S. To address such questions, we applied computationally enhanced quantitative phase microscopy (ceQPM) to populations of cultured human cell lines, which enables highly accurate measurement of cell dry mass of individual cells throughout the cell cycle. From these measurements, we have evaluated the factors that contribute to maintaining cell mass homeostasis at any point in the cell cycle. Our findings reveal that cell mass homeostasis is accurately maintained, despite disruptions to the normal G1/S machinery or perturbations in the rate of cell growth. Control of cell mass is generally not confined to regulation of the G1 length. Instead mass homeostasis is imposed throughout the cell cycle. In the cell lines examined, we find that the coefficient of variation (CV) in dry mass of cells in the population begins to decline well before the G1/S transition and continues to decline throughout S and G2 phases. Among the different cell types tested, the detailed response of cell growth rate to cell mass differs. However, in general, when it falls below that for exponential growth, the natural increase in the CV of cell mass is effectively constrained. We find that both mass-dependent cell cycle regulation and mass-dependent growth rate modulation contribute to reducing cell mass variation within the population. Through the interplay and coordination of these 2 processes, accurate cell mass homeostasis emerges. Such findings reveal previously unappreciated and very general principles of cell size control in proliferating cells. These same regulatory processes might also be operative in terminally differentiated cells. Further quantitative dynamical studies should lead to a better understanding of the underlying molecular mechanisms of cell size control.

## Introduction

The size distribution of a population of proliferating cells is accurately maintained over many generations, despite variability in the growth rate and the duration of the cell cycle in individual cells, as well as the imprecision in the equipartition of daughter cells at mitosis. Each of these factors is known to contribute to a dispersion in cell size within a population [1]. It has long been evident that there must be some “correction” mechanism that would act within individual cells to counteract the combined effects of all the sources of random variation and thereby ensure a stable size distribution in the population over many generations [2]. Studies on mammalian and yeast cell size up to now have focused on 1 attractive and plausible mechanism for size homeostasis: a dependence of the G1 length inversely with cell size. Theoretically, such a mechanism should allow small cells to “catch up” with larger cells by spending a longer time growing in the G1 phase. Such a process would be expected to reduce cell size variation by normalizing size at the point of S phase entry [2–9]. Several molecular players in this process have been suggested, such as the dilution of retinoblastoma (Rb) protein [6,9,10] and the activation of p38 MAPK kinase [11,12]. However, such a mechanism, while attractive for its simplicity, cannot in principle fully explain the constancy in the cell size distribution over many generations. Specifically, if G1 length regulation were the only operative mechanism, cells would have no way to anticipate the random variation introduced during the subsequent nonG1 cell cycle phases, a period longer than G1 in most proliferating cell types. Nevertheless, most proliferating cell populations, regardless of their surrounding environment and genetic background, manage to achieve highly accurate size homeostasis [13].

In 1985, Zetterberg and colleagues reported that the variation of G1 length in mouse fibroblast cells accounted for most of the variation in cell cycle length when cells switched from quiescence to proliferation [14]. However, a later study in several cell lines found the G1, S, and G2 phase lengths had comparable variability and were all positively correlated with the cell cycle length in normal cycling populations [15], implying a dependency of cell cycle phase lengths on cell size outside of G1. Furthermore, regulation of the S and G2 lengths is known to make a contribution to size homeostasis in lower eukaryotic organisms, such as budding and fission yeasts [16–18]. However, evidence of size-dependent regulation outside of G1 has seldom been reported in mammalian cells [4,7]. Little is known about whether the nonG1 phases play an appreciable role in maintaining mammalian cell size homeostasis or whether variation in cell size introduced in the nonG1 phases is somehow fully compensated at the next G1/S transition.

An alternative approach for regulating cell size, other than regulating it at S phase entry or in the length of other cell cycle phases, would be to regulate cell growth [1,19]. A few studies have suggested various types of size-dependent growth rate modulation in cultured cells. For example, Cadart and colleagues found that the slope of volume growth rate versus cell volume decreases for large cells at birth [7]; Neurohr and colleagues found that volume growth rate slows down in excessively large senescent cells [20]; and Ginzberg and colleagues found that nuclear area, an approximate proxy for cell size, is negatively correlated with growth rate at 2 points during the cell cycle [8]. Though such observations have been noted, there has been little said about their quantitative importance. Furthermore, it is hard to evaluate the various types of growth modulation, as they were discovered in different systems using different physical proxies for cell size, such as cell volume and nuclear area. Hence, little can be concluded about whether these processes coexist in the same cell, are specific to certain cell types, or are only reflected in certain types of cell measurement. Compared to studies on cell cycle control, cell growth control has received little attention.

In keeping with a previous study in bacteria [21], we wish to distinguish between “size control” and “size homeostasis.” We will use the term “size control” to refer to the regulation of the mean size, such as when the mean size in a population of cells responds to a change of environment or when cells differentiate into a different cell type; whereas, we reserve the term “size homeostasis” for the control of the variance around the mean size of a population in a defined steady-state condition. Though these 2 processes may turn out to be mechanistically related, we cannot assume that they share the same mechanism. In this study, our focus is on the less well studied but perhaps more common process of size homeostasis. We used cultured cell lines because primary cells can take a very long time to reach a stable cell size in culture, whereas cell lines are much more stable and reproducible. Furthermore, cell lines have been well characterized; hence, observations from different laboratories can be readily compared and experiments can be easily replicated. Finally, we expect that size regulation would occur in all cell types, normal and transformed, embryonic and differentiated. Like other general cellular mechanisms, such as mitosis, DNA replication, and protein secretion, it is highly likely that underlying general mechanisms are conserved. To test this generality, we have studied size regulation during the cell cycle in several human cell lines of diverse origins, cultured under different conditions.

Cell size can be expressed either in terms of mass or volume. Cell volume tends to be a more passive response than mass to the mechanical and osmotic conditions occurring during the cell cycle and differentiation [22–25]. Hence, we have chosen to focus on cell mass homeostasis. There are excellent experimental means to measure cell mass in suspension culture [26], but it is much harder to measure cell mass accurately when cells are attached to a substratum, which is closer to the physiological context for most mammalian cell types. This single experimental limitation has thwarted the study of cell mass homeostasis and growth rate control in the most well-studied systems. Measuring the mass of a single cell on a culture dish accurately is surprisingly difficult. Furthermore, determining the growth rate from the time derivative of the mass is even more challenging [27,28]. The study of cell mass growth rate regulation in attached cells with sufficient precision to distinguish between different models of growth control required the development of new methods. To this end, we recently developed computationally enhanced quantitative phase microscopy (ceQPM), which measures cell dry mass (the cell’s mass excluding water) by the refractive index difference between cell and medium to a precision of better than 2% [29]. To describe statistically significant features of cell mass and growth rate regulation, we tracked single-cell growth and the timing of cell cycle events at a scale of thousands of cells per experiment. Using this improved technology, we could investigate the process of cell mass accumulation relative to cell cycle progression throughout the cell cycle. From these improved measurements, we could derive new understandings of cell mass homeostasis during the cell cycle in several cultured cell lines. The results challenge existing theories of cell mass (or, more colloquially, cell size) homeostasis and suggest further mechanistic experiments.

## Results

### Cell mass variation is tightly controlled and largely independent of the state of the G1/S circuitry

“Cell mass homeostasis” can be strictly defined as the maintenance of a stable distribution of cell mass over generations in a population of proliferating cells. Expressed mathematically, at homeostasis, the coefficient of variation (CV) of cell mass at division, *CV*_*d*_, should be lower than the CV of cell mass at birth, *CV*_*b*_. And, the two should fulfill the equation adapted from Huh and colleagues [30]:

$${CV}_{b}^{2}={CV}_{d}^{2}+Q^{2},$$ (Eq 1)

where *Q* denotes the partition error, with $Q^{2}=\frac{<{\left(m_{1}-m_{2}\right)}^{2}>}{{{<m}_{1}+m_{2}>}^{2}}$; *m*_1_ and *m*_2_ are the birth masses of the 2 daughter cells of the same mother cell, respectively. By monitoring the proliferation and growth of HeLa cells by ceQPM, we found that the cells were indeed at such a homeostatic state, as the difference between the left- and right-hand sides of Eq 1 was negligible (S1 Fig).

**Fig 1:**
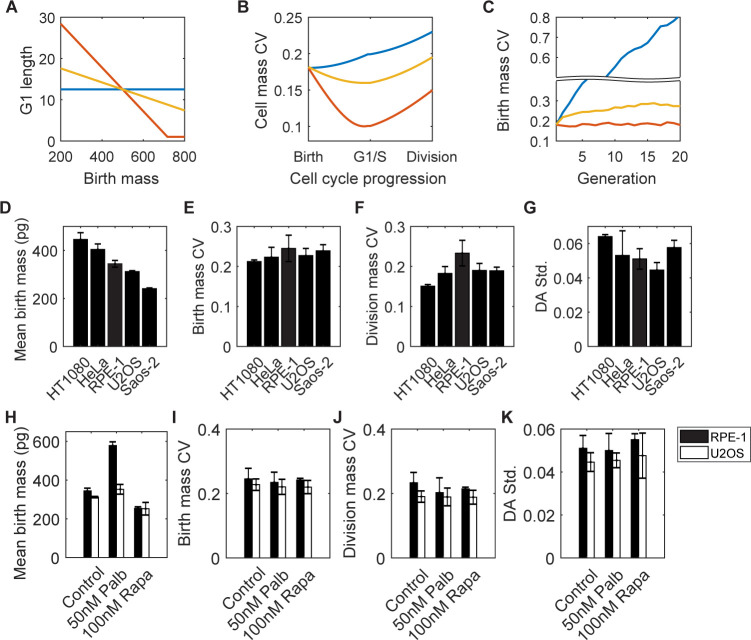
Cell mass variation is tightly controlled in mammalian cell lines and is robust to perturbations in G1/S regulation or growth rate. (A–C) An abstract model of cell mass homeostasis at different G1 regulation strengths, represented by the slope of G1 length vs. birth mass correlation. The corresponding model and simulation parameters are in the Section 1 in S1 Text. In the model, we assume cells grow exponentially, and the G1 length control is the only mechanism to reduce cell mass variation. (A) Correlations between G1 length and birth mass. Blue: no G1 length control; red: with strong G1 length control; yellow: with weak G1 length control. (B) Cell mass CV changes with cell cycle progression during 1 cell cycle with the corresponding G1 length regulation in (A). (C) Birth mass CV changes across generations with the corresponding G1 length regulation in (A). (D–G) The mean birth mass (D), birth mass CV (E), division mass CV (F), and DA std. (G) for different cell lines. (H–K) The mean birth mass (H), birth mass CV (I), division mass CV (J), and DA std. (K) for RPE-1 and U2OS cells in normal culture medium, medium with 50 nM palbociclib, and medium with 100 nM rapamycin at cell mass homeostasis. Error bars in (D–K) indicate the standard deviation of 3 or more measurements. The data underlying this figure and the scripts used to generate the plots are available on the Open Science Framework at osf.io/3kyvw. CV, coefficient of variation; DA std., standard deviation of Division Asymmetry.

To explore this homeostasis further, we considered an abstract model of how the cell mass variation of a cell population evolves with cell cycle progression (Section 1 in S1 Text). If there were no operative controls and cell mass grew exponentially ($\frac{dm}{dt}=\alpha m$) (Fig 1A), the cell mass CV would be expected to increase super-exponentially as the cells traverse the cell cycle due to the variation of the growth exponent, *α*, among cells (Fig 1B). Furthermore, the variation in cell cycle length and the partition error would further contribute to the cell mass variation (quantified by the birth mass CV) at each generation (Fig 1C). To maintain cell mass homeostasis, these accumulated discrepancies must be offset by a reduction of variability by some processes during the cell cycle. If, as suggested in both in vitro and in vivo systems [4,6], the G1/S checkpoint were the principal “size control checkpoint” (Fig 1A), we would expect the reduction in cell mass variation to occur before or at the G1/S transition. The cell mass CV would then be expected to increase super-exponentially after G1/S due to the lack of any operable size control processes in the nonG1 phases. Therefore, the CV reduction before G1/S would have to greatly undershoot the birth mass CV to anticipate and compensate for the cell mass variability that would accumulate during the nonG1 phases (Fig 1B). If the G1/S control were weakened by genetic mutation or pharmacological perturbation (Fig 1A), the reduction in cell mass CV before G1/S would be expected to decrease, and the uncorrected error would cause an increase in the division mass CV (Fig 1B). Such a population would eventually reach a new homeostatic state with higher birth and division mass CVs in order for Eq 1 to be fulfilled (Fig 1C). Therefore, the birth mass CV at homeostasis can be used as an indicator of the stringency of the control on cell mass homeostasis.

To investigate how different forms of G1/S control might affect cell mass homeostasis, we compared various human cancer cell lines, each with different G1/S deficiencies, and RPE-1, a cell line with a wild-type G1/S transition [7,12,31] (S1 Table). To evaluate the stringency of the control mechanisms regulating cell mass homeostasis, we measured the birth and division mass CVs of live cell populations from short-term videos using ceQPM. We define the Division Asymmetry, $DA=\frac{m_{1,2}}{m_{1}+m_{2}}$, where *m*_1_ and *m*_2_ represent the birth masses of the 2 daughter cells, and *m*_1,2_ denotes the mass of either of the daughter cells. For a population that divides with perfect symmetry, the distribution of *DA* should be precisely at 0.5 without any dispersion. But if either daughter cell were larger or smaller than half the mother cell mass, its *DA* would deviate from 0.5. The standard deviation of *DA* (DA std.) quantitatively represents the fidelity of cytokinesis, and it is more commonly used than the partition error *Q* in Eq 1 [17,32]. Despite the considerable variation in cell mass across the different cell lines (the mean birth mass of the largest cell line, HT1080, is 1.85-fold greater than the smallest cell line, Saos-2) (Fig 1D), the difference in birth mass CV is less than 15% for each cell line (Fig 1E); the division mass CV and DA std. for these cell lines were also comparable (Fig 1F and 1G). Note that the measurement error of ceQPM is negligible (less than 2%) compared to the birth and division mass CVs.

To assess the robustness of the birth mass CV to perturbations in the G1/S transition, we perturbed G1/S regulation in both RPE-1 and U2OS cells using a well-characterized CDK4/6 inhibitor, palbociclib [33]. Although U2OS cells have intact Rb proteins, which have been reported to govern the G1/S transition [4,6,34], they carry deficiencies in other G1/S regulators (S1 Table) and are much less sensitive to palbociclib than RPE-1, which has intact G1/S circuitry (S2A and S2B Fig). Both cell lines were examined at a low dose of palbociclib, where there was a delay in G1/S but no arrest of the cell cycle (11). We measured the dry mass of RPE-1 and U2OS cells after being cultured for more than 1 week in palbociclib, at which point the mass distribution of each cell line had reached a new steady state. It had been shown previously that a low dose of palbociclib weakens the negative correlation between birth size and G1 length (like the yellow curve in Fig 1A) [11]. Thus, if G1 regulation were essential for cell mass homeostasis, we would expect the birth mass CV to increase with palbociclib treatment (like the yellow curve in Fig 1C). Surprisingly, although the mean mass at birth had increased by 1.68-fold and 1.13-fold, respectively, in RPE-1 and U2OS cells (Fig 1H), the birth mass CV for either cell line hardly changed and in fact slightly decreased (a 4% and 3% reduction for RPE-1 for U2OS cells, respectively) (Fig 1I). Similarly, the division mass CV and the standard deviation of Division Asymmetry, DA std., also hardly changed after exposure of both cell lines to palbociclib (Fig 1J and 1K). These very small changes in mass CVs indicate that the control of mass homeostasis still operates accurately, despite strong perturbation of the G1/S transition.

Since disruption and delay of the cell cycle at G1/S did not appear to affect mass homeostasis, we examined the inhibition of cell growth for effects on cell mass variability. We used rapamycin, a specific inhibitor of mTOR [35], which has pervasive knock-on effects on protein synthesis and degradation [36]. When RPE-1 and U2OS cultures were exposed to rapamycin, the steady-state birth mass decreased by 27% and 20%, respectively (Fig 1H). However, there were no significant changes in the birth mass CV, division mass CV, or DA std. (changes less than 8% were observed) (Fig 1I–1K). Therefore, it appears that mass homeostasis is strongly buffered, even when mass is greatly perturbed.

### Cell mass variation is regulated throughout the cell cycle

**Fig 2:**
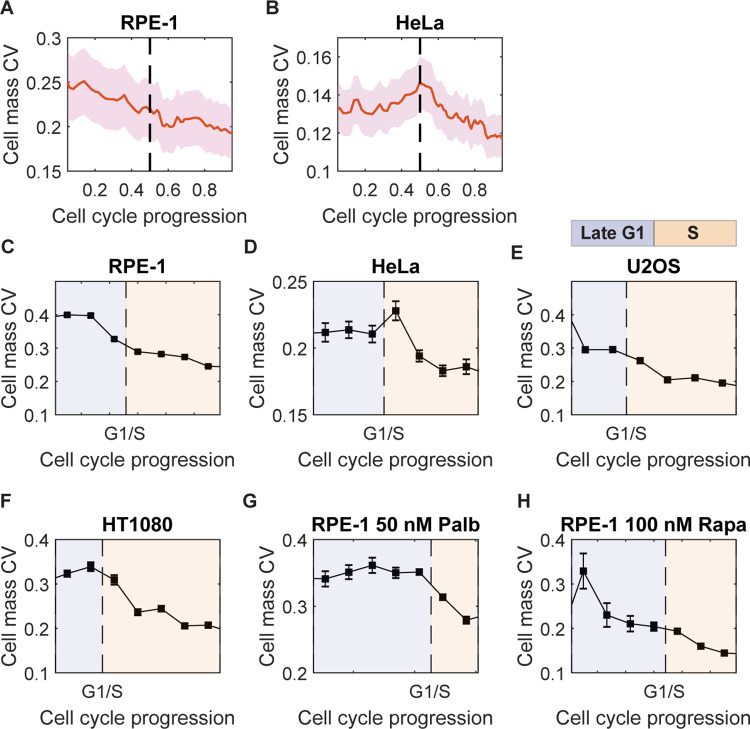
Cell mass variation is regulated throughout the cell cycle. (A, B) Cell mass CV change with cell cycle progression measured in live RPE-1 (*n* = 89) (A) and HeLa cells (*n* = 223) (B). The red solid lines denote the cell mass CV of the population; the pink shadows show the 95% confidence interval; the dashed line indicates the G1/S transition. (C–H) The profiles of how cell mass CV changes with cell cycle progression at cell mass homeostasis measured in fixed RPE-1 (C), HeLa (D), U2OS (E), and HT1080 (F) cells, as well as RPE-1 cells that had reached the new cell mass homeostasis with 50 nM palbociclib (G) or 100 nM rapamycin (H). The cell cycle stages were identified by DNA content and log(mAG-hGeminin) as illustrated in S4B–S4F, S4H, and S4J Fig; the late G1 and S phases are indicated by areas shaded in purple and orange, respectively; error bars are the standard error of CV, ($CV/\sqrt{2n}$), where *n* is the cell number at the corresponding cell cycle stage (*n* > 135 for all conditions). The data underlying this figure and the scripts used to generate the plots are available on the Open Science Framework at osf.io/3kyvw. CV, coefficient of variation.

Using ceQPM, we can now ask at what points during the cell cycle variation in cell mass occurs and at what points it is suppressed. We used the CV as a metric of cell mass variation and measured it throughout the cell cycle in live RPE-1 and HeLa cells. To correlate the CV with the state of the cell cycle, we utilized fluorescently tagged geminin degron as the cell cycle marker. Geminin is a protein that regulates DNA replication. Possessing a destruction sequence like cyclin B, geminin is degraded precisely at mitosis and begins to accumulate precisely at the G1/S transition (S3A Fig) [37]. We aligned individual cell mass trajectories (S3B Fig) by normalizing the length of the G1 segment to 0–0.5 and that of the nonG1 segment to 0.5–1 and then calculated the CV of these normalized cell mass trajectories with cell cycle progression. In RPE-1 cells, the cell mass CV was found to be reduced throughout the cell cycle (Fig 2A), whereas in HeLa cells, the cell mass CV increased in the G1 phase before declining in the nonG1 phases (Fig 2B). Neither cell line exhibited a minimum cell mass CV at the G1/S transition, as would be predicted by conventional G1 length control models (Fig 1B).

To examine the regulation of cell mass variation further in various cell lines and under different conditions, we calculated the cell mass CV profile as a function of cell cycle progression from fixed cells, which provided much higher throughput than our live cell measurements. Using ergodic rate analysis (ERA) (38), we defined a cell cycle mean path and divided it into 13 to 14 segments evenly spaced in time, based on measurements of DNA content and fluorescently tagged geminin degron. We applied this analysis to hundreds of thousands of fixed cells (S4A Fig). By definition DNA replication occurs exclusively in the S phase, whereas geminin accumulation starts at the G1/S transition (S4B–S4E, S4H, and S4J Fig) [38]. Though these 2 markers provide good resolution in late G1 and S phases, they have poor temporal resolution in the early G1 and G2-M phases due to inaccuracy in cell cycle stage identification (S4F Fig). Therefore, we focused our analyses exclusively on the cell mass CV in the late G1 and S phases, employing large numbers of fixed cells.

We applied this approach to 4 cell lines: RPE-1, HeLa, U2OS, and HT1080. The cell mass CV profiles in fixed RPE-1 and HeLa cells (Fig 2C and 2D) were similar to what we had previously found in the live cell trajectories (Fig 2A and 2B), further validating the use of fixed cells to extract cell mass CV profiles. We found that in RPE-1 and U2OS cells, the cell mass CV declined in late G1 (Fig 2C and 2E), as would be expected from conventional models where regulation of the G1 length is thought to be the sole means for normalizing cell size. However, we were surprised to find that the CV of cell mass then continued to decrease progressively through S phase. Most strikingly, in HeLa and HT1080 cells, there was virtually no reduction in cell mass CV in late G1; the major decrease only took place in S phase (Fig 2D and 2F). These quantitative differences in cell mass CV profiles may depend on the status of the G1/S circuitry in these cell lines (S1 Table). These observations are completely at odds with the G1/S transition playing the dominant role in cell size control, although it may remain a critical point for cell cycle regulation [1,19,34]. Note that the decrease in cell mass CV cannot be explained by a reduction in noise because even if noise went to zero at some point, the CV would remain at its previous value. We believe that a very strong conclusion can be drawn from these phenomenological measurements: there must be feedback between cell mass and cell growth rate or between cell mass and cell cycle outside of the G1 phase. The effect of this feedback would be to effectively reduce existing variation in the population in nonG1 phases of the cell cycle.

Since palbociclib and rapamycin had little or no effect on the birth and division mass CVs (Fig 1I and 1J), we wondered whether they affected the timing of mass CV regulation during the cell cycle. Consequently, we carefully measured the cell mass CV profiles in fixed RPE-1 cells that had reached new cell mass homeostasis with either drug. Both drugs altered the duration of the cell cycle phases and particularly extended the G1 phase (S2 Table). As we had done above with untreated cells, we computed the cell cycle mean path of treated cells and examined their cell mass CV as a function of cell cycle progression (S4G–S4J Fig). Strikingly, we found that disrupting the G1/S transition with palbociclib led to a slight increase in cell mass CV in late G1, followed by a much greater reduction in cell mass CV during the S phase (Fig 2G). Conversely, inhibiting cell growth with rapamycin caused a greater reduction of cell mass CV in late G1, and the reduction in S phase became smaller (Fig 2H). These results suggest that the regulation of mass CV during S phase can compensate for the mass CV reduction in late G1. Thus, when there is an insufficient or excessive reduction in mass CV in late G1 due to the inhibition of the G1/S transition or growth, respectively, there is a corresponding change in the mass CV in S phase, which acts to maintain the mass CV reduction at division at the same level.

### Feedback by cell mass not only acts on the duration of G1, but also on the durations of S and G2 phases

To investigate further cell mass regulation outside of the G1 phase, we needed to better optimize the resolution of the cell cycle markers we had employed. We therefore adopted 2 cell cycle markers for live cells that bracketed S phase: mAG-hGeminin [37] and mTurquoise2-SLBP [39]. The APC^Cdh1^ substrate, geminin, starts to accumulate in the nucleus at S phase entry [40], whereas the histone mRNA stem-loop binding protein, SLBP, is rapidly degraded at the end of the S phase [41] (S5A Fig). Unlike the conventional PCNA or DNA ligase I markers, which label replication foci during the S phase [42,43], geminin and SLBP are diffusive in the nucleus and more suitable for the relatively low spatial resolution of the QPM camera. With these 2 markers, we could accurately quantify the durations of G1, S, and G2-M phases. Since the duration of M phase is remarkably constant [15], we attributed most of the variation in G2-M duration to the G2 phase itself. We verified that the timing of S phase, as identified by geminin and SLBP, was consistent with the timing of S phase identified by the DNA ligase I foci (S5B and S5C Fig). None of the markers affected the length of any of the cell cycle phases nor did they affect the mass-dependent regulation of the duration of the cell cycle phases (S3 Table). Moreover, the identification of the cell cycle phases (G1, S, and G2-M) using geminin and SLBP exhibited a similar variability in their lengths as those shown using PCNA as a marker of S-phase by Araujo and colleagues [15] (S2 Table). Therefore, we could be confident that the geminin and SLBP markers faithfully reported the cell cycle phase durations and did not change the physiology of these processes.

**Fig 3:**
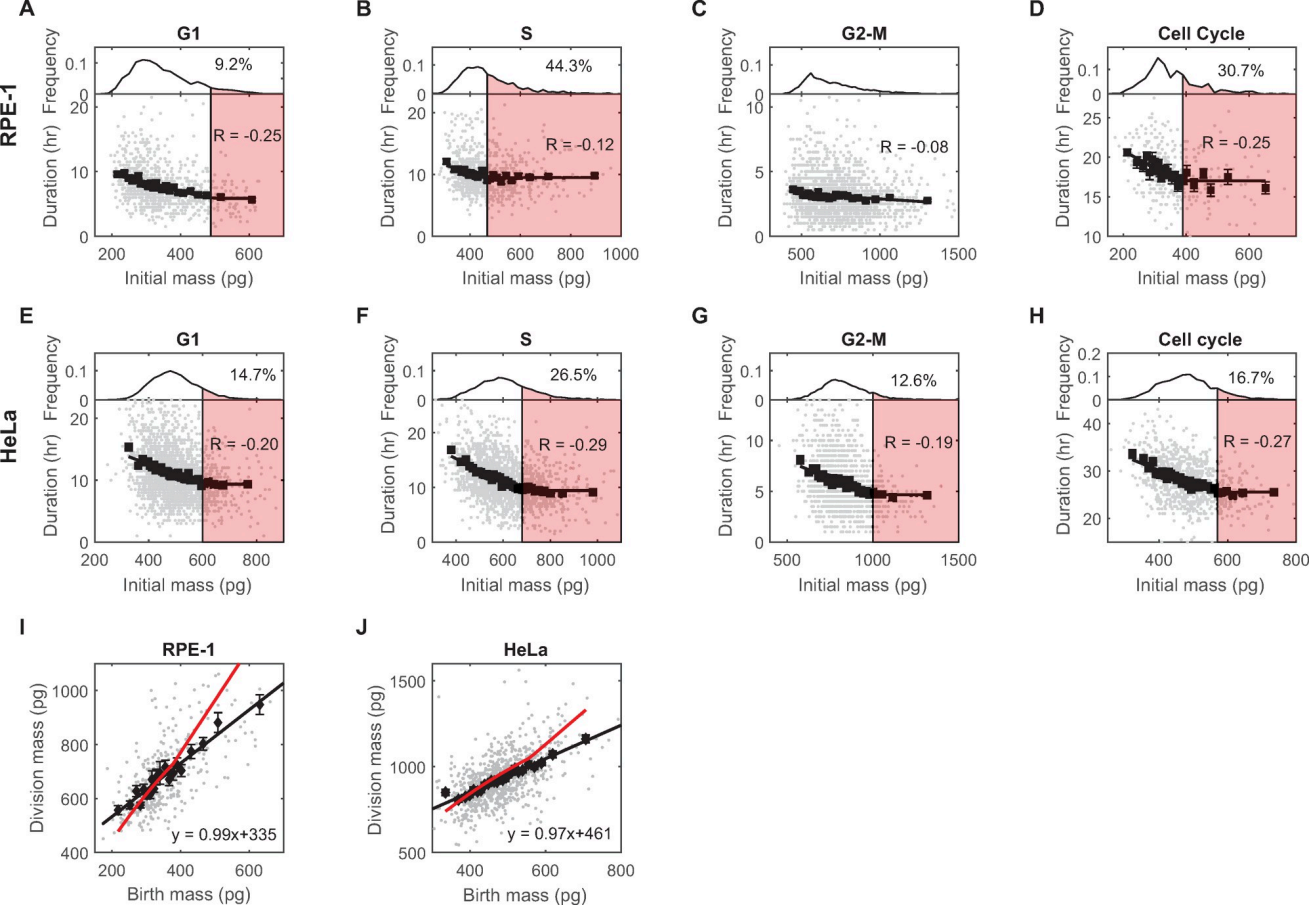
The negative regulation of the durations of the G1, S, and G2 phases by cell mass. (A–D) The correlations between the lengths of the G1 (A), S (B), G2-M phases (C), and the full cell cycle (D) and the initial mass of the corresponding period in RPE-1 cells. The bottom panels indicate the correlation; the top panels are the distributions of the initial mass. Each gray dot in the bottom panels is an observation; R is the correlation coefficient of the gray dots; black squares indicate the average of each cell mass bin; error bars are the SEM; solid black line is the best fit of the black squares (S4 Table). The red shaded area in the top panel indicates the cell mass range that is affected by the minimal cell cycle phase length limit, with the text indicating the percentage of affected cells in the distribution. (E–H) The correlations between the length of the G1 (E), S (F), G2-M phases (G), and the full cell cycle (H) and the initial mass of the corresponding period in HeLa cells. (I, J) The correlations between birth and division masses in RPE-1 (I) and HeLa (J) cells. Each gray dot is an observation; black squares are the average of each cell mass bin; error bars are SEM. The solid black line is the best linear fit of the gray dots; the text indicates the function of the best fit; the red line is the prediction of the best fit in (D) or (H), respectively, assuming that cells grow exponentially (Materials and methods). The data underlying this figure and the scripts used to generate the plots are available on the Open Science Framework at osf.io/3kyvw. SEM, standard error of the mean.

Using this approach, we confirmed the well-established existence of cell size-dependent regulation of G1 length with ceQPM. Consistent with previous findings [3,4,6–8,12], we found that the G1 length was negatively correlated with birth mass in both non-transformed and transformed cell lines, RPE-1 (Fig 3A) and HeLa (Fig 3E), respectively. The correlation was stronger in RPE-1 than in HeLa cells (Fig 3A and 3E and S4 Table). We also investigated the mass-dependent regulation of the durations of both S and G2 phases. S and G2-M phase lengths negatively correlated with the initial mass of the corresponding periods in both RPE-1 and HeLa cells (Fig 3B, 3C, 3F, and 3G). For RPE-1 cells, the correlations of cell cycle phase length with initial mass in S and G2 were weaker than that in G1, yet they were significant (Fig 3A–3C and S4 Table), demonstrating that regulation of cell mass variation can occur through regulating the durations of S and G2 phases in non-transformed cells with an intact cell cycle network, including an intact G1/S transition. This contrasts to the conventional models that would have predicted G1 length to vary inversely with mass while leaving other phases unaffected. We also found in HeLa cells that the negative correlation between cell cycle phase length and mass was much stronger in the S phase, with a correlation coefficient of −0.29, compared to that in the G1 phase, which had a correlation coefficient of −0.20 (Fig 3E and 3F and S4 Table). It is worth noting that although RPE-1 has more stringent G1/S control than HeLa, the overall dependency of cell cycle length on cell mass was not stronger (Fig 3D and 3H and S4 Table). These studies challenge the G1/S checkpoint model, as mass-dependent cell cycle regulation is not restricted to the change in the length of G1 phase as predicted [2,44,45], but rather it is accompanied by changes in the lengths of the other phases of the cell cycle.

Upon closer examination of the binned correlations, we observed a fixed minimum limit for the length of nearly every phase of the cell cycle, as well as the length of the entire cell cycle in RPE-1 and HeLa cells (Fig 3A, 3B, and 3D–3H). These limits are not further reduced in large cells. To summarize these findings, we employ 2 graphical representations for these correlations: a linear model and a bilinear model, comprised of 2 line segments. With these, we fit the binned correlations of mass and cell cycle phase lengths. We found that a bilinear model provided a better fit for all phases of RPE-1 and HeLa cells, with the exception of the G2-M phase in RPE-1 cells (Fig 3A–3H and S4 Table). This graphical relationship implies that regulation of the durations of cell cycle phases cannot effectively control the mass of large cells. To illustrate the impact of the minimal cell cycle length on cell mass variation, we conducted simulations to observe the mean and CV of cell mass within a cell population across generations, while varying the fraction of cells affected by the minimal length limit (Section 2 in S1 Text). The simulations show that as the minimal cell cycle length applies to more and more cells, the homeostatic birth mass CV increases. The system eventually loses homeostasis when the minimal cell cycle length is imposed on more than 40% of the cell population (S6 Fig).

In these experiments, we found that the slope of a graph of birth masses versus division masses was close to 1 in both RPE-1 (Fig 3I) and HeLa cells (Fig 3J), consistent with the adder-like behavior seen previously [7]. The adder model is interpreted as a behavior where cells add a constant amount of mass during the cell cycle regardless of their birth mass. Furthermore, we found in our measurements that each cell cycle phase exhibited an adder-like behavior (S7 Fig), making the full cell cycle a sequential adder. Such behaviors challenge the interpretation that, in mammalian cells, mass regulation arises from a combination of a G1 sizer and a nonG1 timer [19]. Rather, the present findings strongly suggest that each cell cycle phase, except for M phase, contributes to cell mass homeostasis. Moreover, the fitted function of birth mass and cell cycle length correlation cannot fully explain the adder behavior. This is particularly the case for large cells, under the assumption of exponential growth (Fig 3I and 3J). This discrepancy is at least partially due to the existence of a minimal cell cycle phase length. These new results underscore the need for a process of non-exponential growth (or what we term “growth rate modulation”) to maintain cell mass homeostasis in the mammalian cells we have studied, rather than relying solely on processes of cell cycle regulation.

### Mass-dependent growth rate modulation reduces the CV of cell mass during cell cycle progression

The simplest mathematical model for cell growth kinetics, which requires no size sensing or feedback mechanisms, is an exponential model where the growth rate is proportional to size. This has been particularly successful in describing growth in bacteria and can be rationalized by a process of ribosome-dependent ribosome biosynthesis [26,46]. This simple exponential model, however, causes variation in cell size to amplify as cells progress through the cell cycle (Fig 1B and Section 3.1 in S1 Text). Contradicting this model, several studies have found that although large cells generally grow faster than small cells, growth is not precisely exponential in mammalian cells [7,26,29]. Such a lack of exponential growth might in itself lead to a reduction in cell size variation. Various previous studies suggested the dependency of growth rate on cell size changes with cell size and cell cycle stage [7,8,20,47–50]. Recent studies by us and others have found growth rate oscillations [29,51], where a cell alternates between increases and decreases in growth rate.

To explore the dependence of growth rate on cell mass in proliferating cells, we measured the growth rate in a 3-h time window and computed its correlation with cell mass at time zero. We examined how growth rate correlated with cell mass in 18,000 HeLa cells and found that the relation of mass to growth was close to exponential, except for a slight depression for large cells (S8A Fig). Nevertheless, when we segregated the cells into 4 cell cycle phases, we uncovered distinct cell cycle dependencies in such correlations, which were originally masked by pooling all cells for analysis (S8B Fig). An even closer look at the data, with cells categorized into 14 equally divided cell cycle stages, revealed positive-to-negative correlation transitions at various points in the cell cycle (S8C Fig). The slope of the linear relation between cell mass and growth rate for cells in different stages of the cell cycle indicated stronger modulation (greater deviation from the expected slope of exponential growth) in the late G1 and G2-M phases (S8D Fig), consistent with S8B Fig and previous studies [8,38]. However, the proportionality is sub-exponential in most of the cell cycle stages (S8D Fig), suggesting a global process that inherently limits the growth of large cells.

When we investigated the mass versus growth correlations in finely divided cell cycle stages, we found subtle features. Yet, such studies require very large numbers of cells and very accurate growth rate measurements. Coarser cell cycle discrimination leads to a loss of this kind of information on subtle changes in the growth rate, it nevertheless adds greater statistical power to conclusions about overarching aspects of mass-dependent growth regulation. Therefore, there is a practical tradeoff between high cell cycle resolution of the growth analyses and the statistical reliability of the findings. In the following analyses, we aimed for stronger statistical significance and therefore partitioned cells more crudely into the G1 and nonG1 phases, focusing on the most salient features of growth rate modulation. This level of resolution was sufficient to reveal previously undiscovered features, which serve to correct our current understandings.

**Fig 4:**
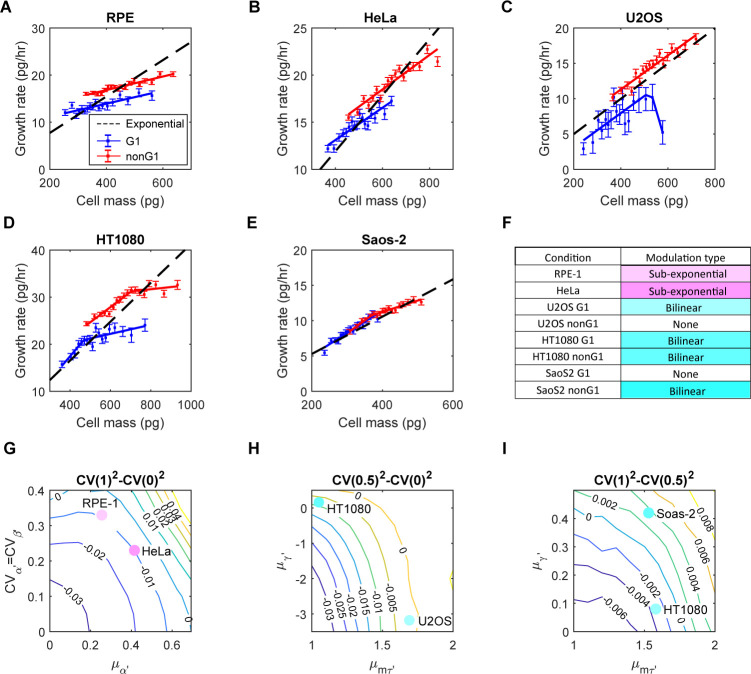
Growth rate dependence on mass differs in different cell lines, and growth rate modulation can effectively reduce cell mass CV during the cell cycle. (A–E) Correlations between cell mass and growth rate in the G1 (blue) and nonG1 (red) phases for RPE-1 (A), HeLa (B), U2OS (C), HT1080 (D), and Saos-2 (E) cells. Filled squares represent the median growth rate of each bin; error bars show SEM. The black dashed lines indicate the expected behavior for exponential growth. The solid blue and red lines are the best fit of the filled squares (S5 Table). (F) The observed conditions were categorized into 3 types: sub-exponential, bilinear, and no modulation. (G) Contour plot illustrating the change in cell mass CV during the entire cell cycle for SE growth rate modulation, as a function of the mean and CV of *α*′ and *β*′, obtained from numerical simulations (Section 3.1 in S1 Text). (H, I) Contour plots illustrating the change in cell mass CV during the G1 (H) and nonG1 phases (I) for BI growth rate modulation, as a function of the means of *γ*′ and $m_{\tau }^{\prime }$, obtained from numerical simulations (Section 3.2 in S1 Text). These simulations assumed a 20% CV in *α*′. Solid circles in (G–I) indicate the corresponding positions in the contour plots when adopting parameter values from the experimental data. The data underlying this figure and the scripts used to generate the plots are available on the Open Science Framework at osf.io/3kyvw. BI, bilinear; CV, coefficient of variation; SE, sub-exponential; SEM, standard error of the mean.

Measuring the correlation between cell mass and growth rate in 5 different cell lines, we found that each cell line behaved somewhat differently. In RPE-1 cells, growth was proportional to cell mass, but the proportionality was much less than exponential, with a significant nonzero intercept (Fig 4A). In HeLa cells, the proportionality between growth and cell mass is much closer to, but slightly less than exponential in both G1 and nonG1 phases (Fig 4B). The observed mass versus growth correlations in short-term measurements in RPE-1 and HeLa cells were consistent with their long-term growth trajectories (S9 Fig), showing nearly linear growth in RPE-1 and a slight deviation from exponential growth in HeLa cells. Therefore, we could confirm that the observed deviation from exponential growth is not due to inspection or sampling bias caused by the short-term measurement [52], but truly signifies the inherent growth law of the cells. In U2OS cells, the correlation was close to exponential for all cells in nonG1 and most cells in G1 phase, but it was abruptly negative for the 15% largest cells in G1 phase (Fig 4C). In HT1080 cells, growth was close to exponential for small cells but transitioned to nearly linear growth in large cells during both G1 and nonG1 phases (Fig 4D). A bilinear model provided a significantly better fit than a simple linear model for cells in the nonG1 phase, indicating the significance of this transition in mass versus growth correlation as cells became larger (S5 Table). In Saos-2 cells, growth was exponential except for a slight deviation for large cells in nonG1 phase (Fig 4E). Taken together, these results indicate that the mathematical description of growth rate is not simply exponential in the cell lines we have investigated, and that different cell lines display different characteristics of mass dependency at different phases of the cell cycle.

To better compare the behaviors of different cell lines, we normalized the mass versus growth correlations, using the means of birth mass and cell cycle length (S6 Table). Since DNA copy number affects the correlation intercepts (Fig 4A–4D), we focused solely on the slope of the correlations. We could distinguish 2 general types of growth rate modulation (Fig 4F and S6 Table). In the first type, growth is linearly related to cell mass, but with a slope lower than exponential growth (RPE-1 and HeLa). We refer to this as sub-exponential (SE) modulation. In the second type, the slope of the mass versus growth correlation is close to exponential for small cells but becomes less positive or even negative for large cells (U2OS G1, HT1080, and SaoS-2 nonG1). We refer to this as bilinear (BI) modulation. For U2OS cells in the nonG1 phase and SaoS-2 cells in the G1 phase, the correlation slope is not significantly different from exponential growth, suggesting minimal regulation.

Other studies had proposed that growth rate modulation contributes to cell size homeostasis [1,7,8,19,38]. However, most of these claims were speculative and lacked sufficient quantitative support. The work by Cadart and colleagues in 2018 stands out as an exception, as it quantitated the correlation between birth size and growth rate [7]. Accurate and quantitative correlations between growth rate and cell size are essential for a thorough assessment of the impact of growth rate regulation. Nevertheless, due to the scarcity of high-quality experimental data, most theoretical investigations into cell size homeostasis have disregarded growth rate regulation completely and focused solely on the regulation of cell cycle length, often assuming exponential growth [53–56]. In this study, we addressed this gap in previous studies by investigating theoretically whether the types of growth rate modulation we observed could effectively reduce cell mass variation. Using stochastic models and simulations, we focused on the influence of growth rate modulation and growth rate noise on the cell mass CV over 1 cell cycle. Initially for convenience, we assumed that all cells divided at the same cell cycle length. Subsequently in more comprehensive models, we incorporated cell cycle regulation and noise, as discussed in a later section.

In the absence of any growth rate modulation, we might imagine that cell mass should accumulate exponentially, as has been found in bacteria [26]. This would cause the cell mass CV to increase super-exponentially due to stochastic variation in growth rate (Section 3.1 in S1 Text). When growth rate modulation is in the SE form (Fig 4F and S6 Table), the slope of the correlation between cell mass and growth rate is lower than that of exponential growth. This can be described by the equation: $\frac{dm^{\prime }}{dt^{\prime }}=\alpha^{\prime }m^{\prime }+\beta^{\prime }$, where the growth rate is composed of 2 terms: *α*′*m*′ represents the part of growth rate proportional to cell mass, whereas *β*′ represents the part independent of cell mass. Here, *m*′ and *t*′ are the cell mass and cell cycle progression time normalized by the means of birth mass and cell cycle length, respectively (Section 3.1 in S1 Text). According to the definition of sub-exponential growth, the mean of *α*′ is smaller than *ln*2 and greater than 0, and the mean of *β*′ is determined by *α*′ when assuming that the mean division mass is twice the mean birth mass, a requirement for maintaining mass homeostasis. For simplicity, we first assumed that *α*′ and *β*′ have the same CV, but we also examined how the CV of either parameter affected the results in the Supporting information (S10 Fig).

During the initial stages of the cell cycle, the cell mass CV consistently decreases, with the rate of decrease negatively correlated with the mean of *α*′ and independent of the CVs of *α*′ and *β*′ (S10A, S10C, and S10F Fig and Section 3.1 in S1 Text). As the cell cycle progresses, the rate of mass CV reduction slows down, and the mass CV may even increase during the later period of the cell cycle (S10B, S10D, and S10G and Section 3.1 in S1 Text). The overall change in the cell mass CV throughout the cell cycle depends on both the mean of *α*′ and the CVs of *α*′ and *β*′. The smaller mean of *α*′ and lower CV of *α*′ and *β*′ result in a more significant reduction in the cell mass CV (Figs 4G, S10E, and S10H). In summary, growth rate variability (characterized by the CVs of *α*′ and *β*′) amplifies cell mass variation, while strong growth rate modulation (small *α*′) can reduce cell mass variation throughout the cell cycle.

To assess whether growth rate modulation in RPE-1 and HeLa cells can cause cell mass CV reduction throughout the cell cycle, we derived the parameters from the experimental data. The mean of *α*′ was determined based on the mean correlations in Fig 4A and 4B (S6 Table). To estimate the variation in *α*′, we used long-term live-cell growth trajectories. The CV of *α*′ was found to be independent of cell mass (S11A and S11B Fig). The variability in *α*′ arises from 2 sources: stochastic partitioning of cellular contents during cell division (intercellular variability) and intrinsic fluctuations in biochemical reactions (intracellular variability) [57]. The former, determined at birth, is a major contributor to cell mass variation, while the effect of the latter gradually cancels out over time, exerting minimal impact on cell mass variation. Therefore, we focused on the intercellular variability and estimated it by calculating the variation among the means of individual growth trajectories (S11C Fig). The CV of *α*′ was estimated to be 0.33 for RPE-1 and 0.23 for HeLa cells, respectively. It is challenging to isolate the variation in *β*′ from measurement error, thus we conducted simulations with *β*′ having the same CV as *α*′ or with the CV of *β*′ being equal to zero. Using these parameters, we found that both RPE-1 and HeLa cells could reduce the cell mass CV after 1 cell cycle (Figs 4G, S10E, and S10H). Since the minimal requirement for cell mass homeostasis is to have a lower cell mass CV at division than that at birth, we concluded that growth rate modulation alone is sufficient to maintain cell mass homeostasis in RPE-1 and HeLa cells.

When a plot of growth rate versus mass is in the BI form (Fig 4F and S6 Table), the slope of the mass versus growth correlation is close to exponential for small cells and becomes less positive or even negative in large cells. This can be described by the equation: $\frac{dm^{\prime }}{dt^{\prime }}=\alpha^{\prime }m^{\prime }(m^{\prime }<m_{\tau }^{\prime })+(\gamma^{\prime }m^{\prime }+\alpha^{\prime }m_{\tau }^{\prime }-\gamma^{\prime }m_{\tau }^{\prime })(m^{\prime }\geq m_{\tau }^{\prime })$, where the mean of *α*′ is close to *ln*2 and the mean of *γ*′ is smaller than *ln*2 (Section 3.2 in S1 Text). The first term on the right side of the equation represents the exponential portion of the mass versus growth rate correlation, while the second term describes the part where growth rate modulation takes effect. Here, *γ*′ indicates the strength of modulation and *m*_*τ*_′ signifies the cell mass at which this modulation begins to take effect. Both *γ*′ and *m*_*τ*_′ are normalized by the means of cell cycle length and birth mass, respectively. Our findings indicate that the increase in cell mass CV is primarily driven by the CV of *α*′ (S12A–S12D Fig). Additionally, we investigated the impact of the means of *γ*′, and *m*_*τ*_′ on the change in cell mass CV throughout the cell cycle. We found that the smaller the means of *γ*′ and *m*_*τ*_′, which means the stronger the modulation on growth rate and the more cells it affects, the greater the cell mass CV reduction (Figs 4H and 4I and S12).

To assess whether the growth rate modulation on its own in U2OS, HT1080, and SaoS-2 cells can also lead to a reduction in cell mass CV, we simulated the changes in cell mass CV during the G1 or nonG1 phase using values of *γ*′ and *m*_*τ*_′ obtained from the experimental data. When assuming a 20% CV for *α*′, growth rate modulation was found to decrease the cell mass CV in the G1 phase for U2OS and HT1080 cells (Fig 4H), as well as in the nonG1 phase for HT1080 cells (Fig 4I). However, it was not sufficient to reduce the cell mass CV in the nonG1 phase for SaoS-2 cells (Fig 4I). As the CV of *α*′ increases, the reduction in cell mass CV becomes less pronounced (S12E–S12H Fig). Eventually, all 3 cell lines fail to reduce cell mass CV at a 40% CV for *α*′ (S12G and S12H Fig). Notably, despite U2OS G1 cells exhibiting a greatly negative *γ*′ value, which indicated an exceptionally strong growth rate modulation, its effectiveness in reducing cell mass CV was lower than that of HT1080 G1 cells due to a smaller proportion of affected cells in U2OS, represented by a larger *m*_*τ*_′.

In summary, we found diverse patterns of correlation between cell mass and growth rate in different cell lines, and within the same cell line measured at different cell cycle stages. We developed stochastic models to explore the impact of different mass versus growth correlations on the change in cell mass CV throughout the cell cycle. These models are representations of the data itself and not contrived schemes. They suggest strongly that in many cases sub-exponential growth, either for all cells or even for a subset of cells, can be an effective means of reducing cell mass CV and can ensure cell mass homeostasis.

### Regulation of the cell cycle and regulation of growth rate compensate for each other to maintain cell mass homeostasis

Both mass-dependent regulation of the progression through the cell cycle and mass-dependent regulation of growth rate are used by cells to reduce cell mass variation. To evaluate the relative importance of these processes in maintaining cell mass homeostasis, we have tried to perturb each mechanism individually in RPE-1 cells.

**Fig 5:**
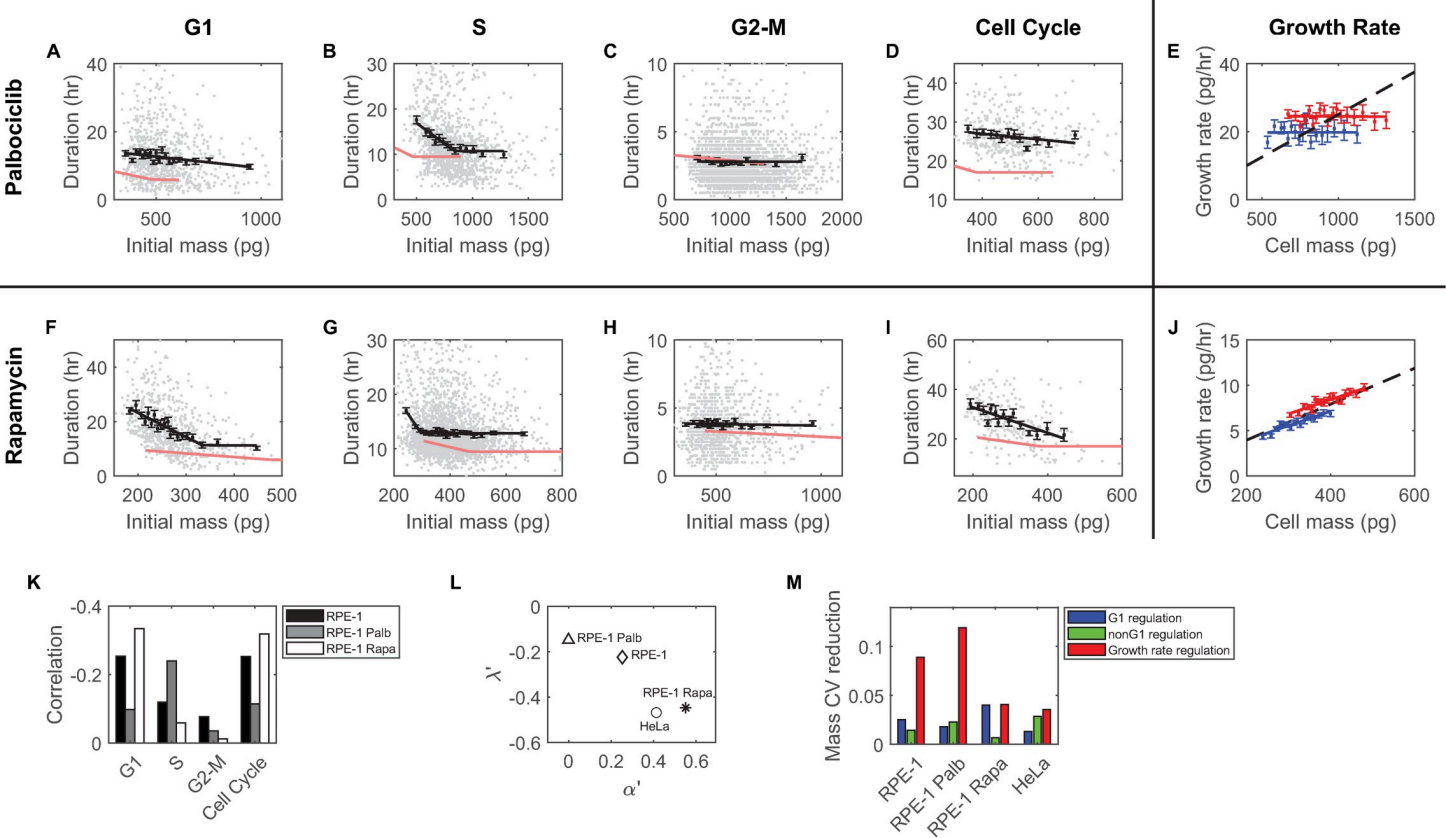
The compensatory roles of mass-dependent cell cycle regulation and mass-dependent growth rate regulation in maintaining cell mass homeostasis. (A–D) The correlations between the lengths of the G1 (A), S (B), G2-M phases(C), and the full cell cycle (D) and the initial mass of the corresponding period in RPE-1 cells treated with 50 nM palbociclib. Each gray dot is an observation; black squares indicate the average of each cell mass bin; error bars are SEM; solid black line is the best fit of the black squares; solid red lines are the corresponding correlations in untreated RPE-1 cells. (E) Correlations between cell mass and growth rate in the G1 (blue) and nonG1 (red) phases for RPE-1 cells treated with 50 nM palbociclib. Filled squares represent the median growth rate of each bin; error bars show SEM. The black dashed lines indicate the expected behavior for exponential growth. The solid blue and red lines are the best fit of the filled squares. (F–I) The correlation between the lengths of the G1 (F), S (G), G2-M phases (H), and the full cell cycle (I) and the initial mass of the corresponding period in RPE-1 cells treated with 100 nM rapamycin. (J) Correlations between cell mass and growth rate in the G1 (blue) and nonG1 (red) phases for RPE-1 cells treated with 100 nM rapamycin. (K) Kendall rank correlations between the duration of indicated cell cycle phase and cell mass at the initiation of the respective phase, in untreated RPE-1 cells, RPE-1 treated with 50 nM palbociclib, and RPE-1 treated with 100 nM rapamycin. (L) The correlation between the normalized slope of birth mass vs. cell cycle length correlation, *λ*′, and the normalized slope of cell mass vs. growth rate correlation, *α*′, depicted for untreated HeLa and RPE-1 cells, as well as RPE-1 cells treated with palbociclib or rapamycin. The values of *λ*′ and *α*′ used in this plot are listed in S7 Table. (M) The contribution of each control mechanism shown as the reduction in the simulated division mass CV when the respective control mechanism is included compared to that without any control mechanisms. Simulation parameters were obtained from experimental data measured in untreated HeLa and RPE-1 cells, as well as RPE-1 cells treated with palbociclib or rapamycin. The data underlying this figure and the scripts used to generate the plots are available on the Open Science Framework at osf.io/3kyvw. CV, coefficient of variation; SEM, standard error of the mean.

To disrupt mass-dependent regulation of G1 length, we slowed entry into S phase using palbociclib, an inhibitor that specifically blocks the activation of Cdk4,6, which is required for entry into S phase [33]. As discussed, low concentrations of palbociclib increased the mean cell mass and, as expected, prolonged the cell cycle length by elongating the G1 phase (Fig 1H and S2 Table). However, once treated cells reached a new homeostatic state, the CV of birth mass remained unchanged compared to untreated cells (Fig 1I). When we analyzed the duration of each cell cycle phase as a function of cell mass, we found a reduced impact of cell mass on G1 phase length coupled with an enhanced impact on S phase length, characterized by the slopes and the correlation coefficients of the correlations between cell mass and the durations of these phases (Fig 5A, 5B, and 5K). Additionally, the mass-dependent regulation of G2 phase was diminished, yet still statistically significant (*p* = 0.0057) (Fig 5C and 5K). These opposite changes in G1 and S phase regulation suggest that the mass-dependent regulation of S phase had effectively compensated for a weakened impact of cell mass on G1 length regulation. Hence, in specific circumstances such as palbociclib treatment, S phase can become the primary period responsible for reducing cell mass variation (Fig 2G). Nevertheless, such compensation ultimately proves insufficient, resulting in a diminished cell mass-dependent regulation of the entire cell cycle length (Fig 5D and 5K). To maintain the birth mass CV at the same level as untreated cells, additional regulation of growth rate is required to further reduce cell mass variation during the cell cycle. Indeed, we found that the correlations between cell mass and growth rate in palbociclib-treated cells were even closer to linear growth compared to untreated cells (Fig 5E), implying a stronger growth rate modulation and a greater reduction in cell mass variation through growth rate regulation. The unchanged CV of birth mass when cells are treated with the G1/S inhibitor, palbociclib (Fig 1I), is a collective result of the interplay between mass-dependent cell cycle regulation and mass-dependent growth rate regulation. Thus, the cell mass CV is maintained despite a significant increase in the mean birth mass (Fig 1H).

In a converse experiment, we specifically perturbed cell growth rate. We treated cells with rapamycin to inhibit mTOR activity. Treatment with rapamycin resulted in an elongation of the cell cycle (S2 Table) and a decrease in mean cell mass (Fig 1H). Similar to the results with palbociclib treatment, rapamycin treatment left the birth mass CV unchanged (Fig 1I). Cell mass-dependent feedback on G1 length was enhanced in the presence of rapamycin (Fig 5F and 5K), while feedback on the S and G2-M phases were weakened (Fig 5G, 5H, and 5K). Additionally, the minimal lengths of all cell cycle phases were slightly increased compared to untreated cells (Fig 5F–5H). In the presence of rapamycin, the cell mass fed back more strongly on the entire cell cycle length, as indicated by the more negative slope and correlation coefficient of the mass versus cell cycle length correlation (Fig 5I and 5K). Furthermore, the relative strengths of correlations between cell mass and cell cycle phase lengths aligned with the reduced cell mass CV in the corresponding phases: for example, cell mass CV was primarily reduced in the G1 phase with rapamycin treatment (Fig 2H), consistent with the strengthened cell cycle regulation in the G1 phase. On the other hand, we found that the slopes of the mass versus growth correlations in both the G1 and nonG1 phases closely resembled that of exponential growth (Fig 5J), suggesting a weaker role of growth rate regulation in maintaining cell mass homeostasis when growth rate is inhibited by rapamycin.

From the experiments described above, mass-dependent cell cycle regulation and mass-dependent growth rate modulation must interact with each other to maintain the birth mass CV at a consistent level even when the G1/S transition or cell growth rate is perturbed, resulting in significant changes in the mean birth mass. After studying the feedback of cell mass on cell cycle length and growth rate under many different circumstances, we felt a need for a new way to compare the response of each under different conditions. We have found it convenient to define a new parameter to represent the strength of this linkage. We utilized the normalized slope of birth mass versus cell cycle length correlation as the parameter *λ*′, which quantifies the strength of mass-dependent cell cycle regulation. The value of *λ*′ is always negative. A more negative value of *λ*′ indicates stronger regulation. Additionally, since the slopes of the cell mass versus growth rate correlations in the G1 and nonG1 phases were similar in RPE-1 and HeLa cells, we found it useful to calculate the average slope of these phases and normalized it by the mean doubling time to represent the strength of mass-dependent growth rate regulation, which we denoted as *α*′. The value of *α*′ is smaller than or equal to *ln*2, which represents exponential growth. A smaller value of *α*′ indicates a greater deviation from exponential growth and thus a stronger modulation of growth rate. We found an inverse correlation between *λ*′ and *α*′ across all the conditions we have investigated (Fig 5L and S7 Table), suggesting a compensatory effect between the regulation of cell cycle and growth rate (i.e., the strengths of these regulatory processes tend to change in opposite directions). For example, when cell cycle regulation was inhibited (e.g., by palbociclib), the modulation of growth rate became stronger, and conversely, when growth rate regulation was inhibited (e.g., by rapamycin), the modulation of cell cycle length became stronger. These findings highlight the compensatory roles played by these 2 processes in maintaining cell mass homeostasis.

To illustrate further the compensatory roles of regulation on cell cycle and growth rate, we developed a stochastic model to simulate changes in cell mass variation throughout the cell cycle (Section 4 in S1 Text). In this model, we considered 3 factors that could contribute to the increase of cell mass variation: variability in cell cycle length, variability in growth rate, and noise in cell mass partition during mitotic division. For simplicity, we only considered intercellular noise as the source of growth rate variability, which is due to stochasticity in the partitioning of cellular contents during cell division, as previously discussed (S11C Fig and Section 3.1 in S1 Text). As control mechanisms, we considered mass-dependent regulation of the duration of G1 and nonG1 phases separately, and we also considered mass-dependent growth modulation throughout the entire cell cycle. We chose all the parameters in this model from our actual experimental data and evaluated the impact of each control mechanism by comparing the cell mass CV at division with and without these control mechanisms. Notably, we observed some discrepancies between the simulated division mass CV, incorporating all 3 control mechanisms, and the values measured in experiments (S8 Table). These may arise from the simplification of variability in growth rate (S11C Fig and Section 4 in S1 Text), which effectively influences cell mass variation (S13 Fig) but is quite challenging to estimate accurately from experimental data. Nevertheless, these simulations largely reflect the relative significance of each control mechanism in maintaining cell mass homeostasis.

The model results indicate that in RPE-1 cells, the regulation of G1 length plays a slightly greater role compared to nonG1 length regulation, but both are overshadowed by the modulation of growth rate (Fig 5M). When the G1/S control is inhibited by palbociclib, the contribution of G1 length regulation slightly decreases, the contribution of nonG1 regulation slightly increases, and the role of growth rate modulation becomes even more dominant (Fig 5M). On the other hand, inhibiting growth with rapamycin leads to an increase in the dominance of G1 length regulation, with its contribution now comparable to that of growth rate modulation, while the impact of nonG1 regulation becomes smaller (Fig 5M). In HeLa cells, the cell mass variation is considerably smaller than that in RPE-1 cells (S8 Table) when not including any control mechanisms, due to the lower variation in growth rate in HeLa cells. It is worth noting that HeLa cells possess a mutated G1/S network. Its ranking of contributions from the 3 mechanisms is similar to the scenario observed in RPE-1 cells treated with palbociclib, which disrupts the G1/S transition. Specifically, in HeLa cells, the contribution of growth rate modulation outweighs that of nonG1 length regulation, which, in turn, outweighs that of G1 length regulation (Fig 5M).

These findings collectively reveal compensatory roles of cell cycle and growth rate regulation in reducing cell mass variation, particularly distinguishing the regulation of G1 length and the regulation of growth rate. Generally, growth rate modulation, rather than cell cycle regulation, is the more dominant mechanism. When one feedback process is hindered, other mechanisms become relatively stronger to maintain cell mass variation at a similar level. Growth rate modulation, rather than cell cycle regulation, consistently plays the predominant role in reducing cell mass CV, regardless of whether or not the cells possess an intact G1/S circuit. In the most extreme case, we studied when the growth rate is inhibited by rapamycin, the contribution of growth rate modulation is on par with that of G1 length regulation. These observations contradict the conventional size control models [1,14,19,44,58–62], which predict that G1/S control is the primary contributor to size homeostasis in mammalian cells.

### Other explanations for how a population of cells might reduce its cell mass variation

We evaluated additional processes that could potentially contribute to the reduction in cell mass CV but were not accounted for in our stochastic model. In principle, any process that affects the likelihood of cell division or cell viability differentially in large and small cells could influence the distribution of cell mass within a population. To estimate the importance of such effects, we examined the rate of cell death and cell cycle arrest through long-term measurements of cell growth and proliferation. During the 48 to 72-h duration of our cell measurements, we defined cell cycle arrest events as instances where a cell remained in the same cell cycle phase while its mass continued to increase throughout the experiment. Furthermore, cell death was identified by a sudden and drastic decrease in cell dry mass, suggesting cell membrane permeabilization.

We found events of cell cycle arrest or cell death in the culture affected no more than 2% of cells in all the conditions that were studied (S9 Table). In particular, neither cell cycle arrest nor cell death occurred frequently enough to contribute significantly to cell mass homeostasis in any of the experiments that we have described. It is worth noting that the remarkably low frequency of cell cycle arrest in cells treated with rapamycin and palbociclib at the drug concentrations used in this study suggests that these drugs at low concentrations do not induce quiescence or senescence at the population level (S9 Table). Furthermore, the concentrations of these drugs did not appear to be toxic enough to cause significant cell death (S9 Table). One intriguing observation was that some large RPE-1 cells treated with palbociclib experienced a partial loss of cytoplasm during mitosis (S9 Table and S1 Movie). This cytoplasmic loss could be attributed to incomplete cortical contraction during mitotic rounding [63]. The amount of mass loss appeared to be random. Notably, these rare events, accounting for approximately 0.5% of cells, did not have a significant impact at the population level on cell mass homeostasis in the presence of palbociclib.

It is worth noting that although these mechanisms were of negligible importance in the specific experimental setting of our study, they might still play a significant role in a tissue setting, for example, during wound healing, regeneration, aging, and/or disease.

### A picture of cell mass homeostasis in proliferating cells

Homeostasis refers to the maintenance of a balance between inherent noise in cellular processes and feedback control mechanisms that correct for them. In proliferating cells, this noise arises from stochastic variation in growth rate, cell cycle length, and cell mass partitioning during mitosis. To reduce cell mass variation, mass-dependent regulation can occur through the control of cell cycle progression, growth rate, or both.

**Fig 6:**
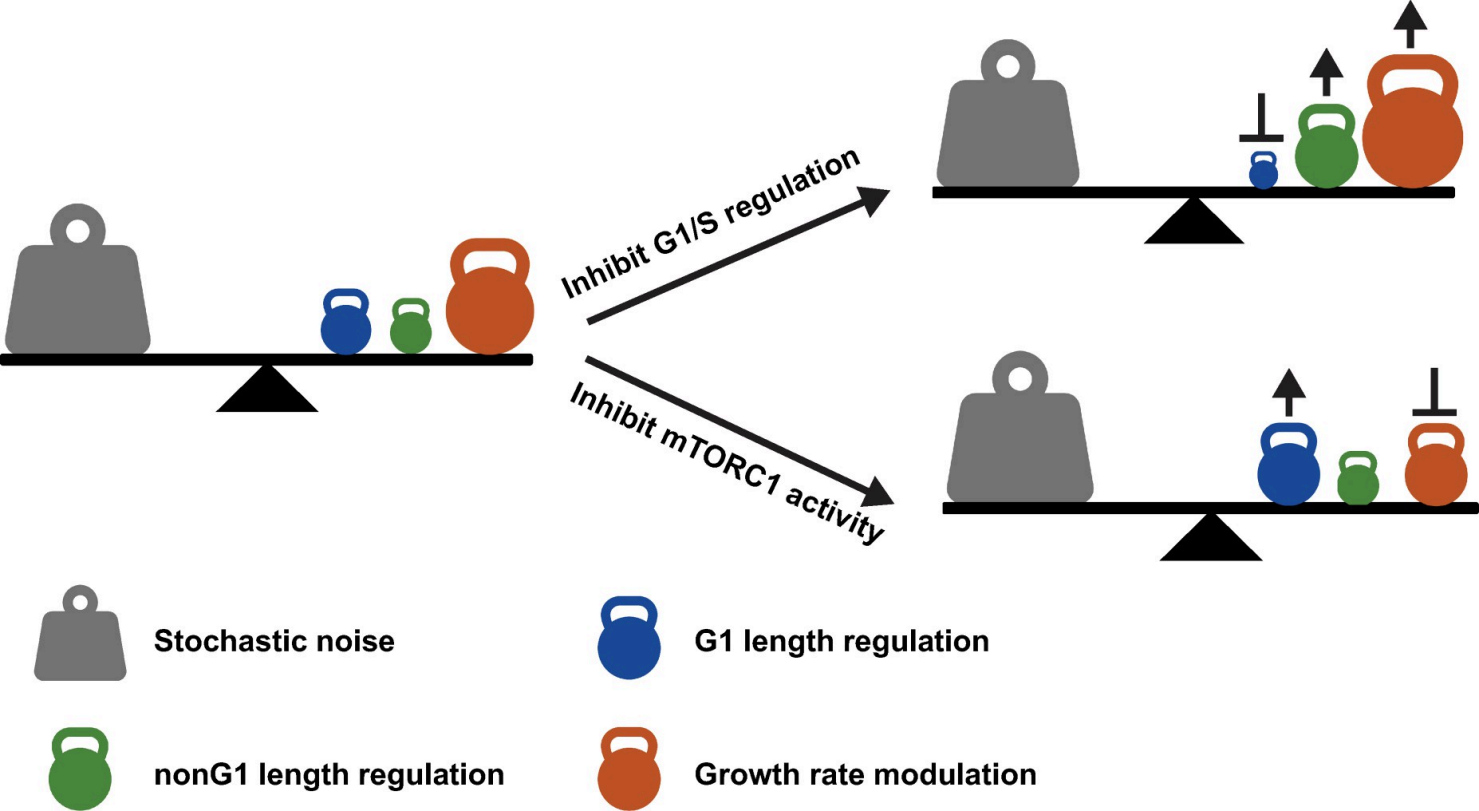
The teeter-totter model of cell mass homeostasis. Cell mass homeostasis requires a balance between stochastic noise and control mechanisms. In unperturbed cells with an intact G1/S circuitry, the weights of control mechanisms from the heaviest to the lightest are the growth rate modulation, G1 length regulation, and nonG1 length regulation. When G1/S control is perturbed, the impact of the G1 length regulation becomes smaller, and the nonG1 length regulation and growth rate modulation become larger to compensate. When the growth rate modulation is suppressed, the G1 length regulation plays a more prominent role in compensating for the reduced impact of growth rate modulation.

To illustrate mass regulation graphically as a balance between noise and control mechanisms, we have depicted the concept of cell mass homeostasis as a “teeter-totter” (Fig 6). Stochastic noise and feedback control mechanisms are represented as opposing forces on either side of the lever’s fulcrum; the sizes of the icons represent the importance of the processes, as determined from the stochastic models (S8 Table). When these effects are balanced, the system reaches a steady state. In cell lines like RPE-1, where the G1/S circuit is intact, the relative importance of the control mechanisms can be ranked from greatest (heaviest on the teeter-totter) to smallest (lightest on the teeter-totter) as follows: growth rate modulation, G1 length regulation, and nonG1 length regulation. A perturbation of the system leads to changes in the stochastic nature of the processes and affects the operation of specific control mechanisms. When this happens, other control mechanisms compensate for these changes and restore the balance. For example, when G1/S control is inhibited, either through pharmacological inhibitors, such as palbociclib, or genetic mutations in the G1/S circuitry, as seen in HeLa cells, the contribution of G1 length regulation is reduced. In response, nonG1 length regulation and growth rate modulation become more significant. Conversely, when growth rate modulation is inhibited, such as by rapamycin, G1 length regulation becomes more important, and growth rate modulation contributes less.

Overall, the teeter-totter of cell mass homeostasis is robustly balanced through the compensatory interactions of these different control processes within the cell. It is likely that the coordination and adjustment of these compensatory mechanisms at the molecular level are crucial for cellular survival under changing conditions. While our understanding of how these mechanisms achieve balance has advanced, further study is needed to elucidate how they coordinate and adapt their compensation at the molecular level to maintain balance under changed conditions and how this plays out in health, disease, aging, etc.

## Discussion

To summarize: in examining cell mass homeostasis, we found that stochastic variation in cell mass in proliferating cells is tightly controlled throughout the cell cycle (Fig 2) via mass-dependent regulation of cell growth rate (Fig 4) and mass-dependent regulation of cell cycle progression (Fig 3). Generally speaking, among the cell lines and cell cycle and cell growth inhibitors that we have employed (including those previously studied and analyzed), we conclude that the G1/S transition does not appear to be a privileged place where cell mass regulation is imposed. Rather mass regulation occurs throughout the cell cycle phases. The compensation that keeps stochastic variation of mass in check emerges from an interplay of these mechanisms and results in effective cell mass regulation. Not only is homeostasis maintained, but it is also maintained at high stringency, as indicated by the narrow distribution of cell mass at birth (Fig 1). Furthermore, cell mass homeostasis is robust to changes in genetic background and is resistant to manipulations of the G1/S transition or perturbation of mTOR activity (Fig 1). The birth size CVs measured in many proliferating bacterial, yeast, mammalian, and plant cells fall in a relatively small range (from 11% to 25%) (S10 Table), which is comparable to the birth weight CV of a human fetus [64]. Although it is not clear whether such strict control is explicitly selected for during evolution or merely a by-product of some other selection [65,66], cell size homeostasis appears to be highly regulated and presumably important. Though we focused on cultured human cell lines in this study, the mechanisms underlying cell size homeostasis, just as the mechanisms underlying the cell cycle itself, are likely to be conserved.

In this study, we utilized ceQPM [29] as a means of measuring cell dry mass, providing a complementary approach to previous studies that focused on cell volume as an indicator of cell size [3,7]. We found that many aspects of the behavior of cell mass, as directly measured by ceQPM, were consistent with studies of cell volume, particularly those reported by Cadart and colleagues, who obtained high-quality cell volume data [7]. For example, in line with their observations, we also identified inverse correlations between initial mass and cell cycle phase duration in both the G1 and nonG1 phases in HeLa cells (Fig 3), the existence of a minimal duration of the G1 phase (Fig 3), the “adder”-like correlation between the birth and division masses (Fig 3), and the coordination between mass-dependent cell cycle regulation and growth rate modulation in maintaining cell mass homeostasis (Fig 5). This consistency is further supported by our recent findings that cell volume usually changes proportionally with cell mass in cultured proliferating cells, except during mitosis, resulting in a narrow distribution of cell mass density [67]. However, we were able to observe more detailed discrepancies in the regulation of mass and volume growth. For example, while Cadart and colleagues reported that volume growth rate is dependent on cell volume at birth [7], we found that mass growth rate is related to cell mass at any point of the cell cycle, and this relationship varies across different cell cycle stages (Figs 4 and S8). Moreover, the noise in mass growth rate appears to affect the slope of the correlation (S11 Fig), in contrast to Cadart and colleagues’ findings of noise primarily impacting the intercept of volume growth rate [68]. These discrepancies may be attributed to inherent differences in the factors affecting mass or volume and the speed and mechanisms by which cells respond to perturbations or fluctuations in mass or volume [69].

Aside from confirming previous discoveries, our findings took a significant step forward in exploring mechanisms underlying cell mass homeostasis. Extensive data collection on large populations of cells was possible thanks to the high-throughput of ceQPM [29]. From these extensive measurements, we derived reliable correlations between cell mass, the durations of cell cycle phases, and the growth rate. We studied these across multiple cell lines and under various pharmacologic perturbations. We were able to fit such data to simple functions (Figs 3, 4, and 5), which facilitated our ability to derive quantitative models. These models, in turn, facilitated our interpretation of the underlying cellular responses. For example, we showed how G1, S, and G2 phases are each under negative regulation by cell mass in both transformed and non-transformed cells (Fig 3). A particularly noteworthy discovery was the identification of a minimum length for each phase of the cell cycle in large cells, which explains the limited impact of cell cycle regulation on very large cells, leaving the underlying process to growth rate modulation (Fig 3). We further demonstrated that growth rate is modulated differently in different cell types or cell lines (Fig 4). Such comprehensive characterization of growth regulation was not previously possible without the extensive and precise measurements of cell mass and growth rate by ceQPM [29]. When we perturbed cells by inhibiting the G1/S transition or suppressing the growth rate (Fig 5), ceQPM enabled us to go beyond the qualitative phenomena observed in previous studies [8,11,12]. It allowed us not only to determine the average changes in cell mass, cell cycle phase duration, and growth rate but also to measure these qualities at the single-cell level, tracking the individual cells over time. This enabled us to derive important quantitative correlation functions. These functions in turn allowed us to write deterministic equations, incorporate stochastic noise, and ultimately develop a stochastic model. With this model, we could estimate the relative weight of each of the regulatory mechanisms employed in maintaining cell mass homeostasis and finally deduce how the weights of these separate mechanisms depend on each other (Fig 5).

One simple finding stands out. It has been generally assumed, and widely cited in review articles and textbooks of biology, that G1 length regulation is the predominant or even the sole mechanism controlling cell size during the cell cycle [1,14,19,44,58–62]. There was always an appeal of this simple mechanism, as it made perturbation of the cell cycle at G1/S the whole process for cell size control. We now can say that this is clearly not the case. Our current highly quantitative studies involving at least hundreds of cells per condition demonstrated that, at least for the cell lines we employed, the impact of G1 length regulation on constraining cell mass CV within a proliferating cell population is much less significant than the modulation of cells’ mass accumulation (growth) rate (Fig 5). This holds true for non-transformed cells with intact G1/S control. Furthermore, even in the presence of growth inhibition induced by rapamycin, the contribution of growth rate modulation to cell mass CV reduction is no less than that of G1 length regulation.

Why would there be size-dependent growth rate regulation if regulation of cell cycle progression were sufficient to control cell size? With so many essential genes in the genome, it seems like a weak argument to claim that having 2 separate mechanisms provides increased security for survival. We propose instead that they serve 2 separate functions. Control of the G1 length might be used primarily to set the cell size for a given cell type. In this view, the G1/S transition is hard-wired into developmental pathways like the MAP kinase pathway or the BMP pathway through proteins like TGFβ. By contrast, control of cell growth might be primarily used for a different purpose: maintaining cell size homeostasis of any given cell type against environmental or stochastic variation. It makes more sense that the targeted mean size of a given cell type is controlled by a few key molecular players downstream of specific hormonal or nutrient signals or cellular differentiation. Those molecular players (such as CDK4/6 or other CDK inhibitors) were described as a cell size “dial” in a previous model by Tan and colleagues [11]. However, once cells are programmed to adopt a defined size in their new state, they would still require a mechanism to maintain size homeostasis around that new mean by buffering against environmental or internal stochastic fluctuation. Consistent with the work presented here (Fig 1) and studies in budding and fission yeasts [13,17], deletion or overexpression of the G1/S inhibitors change the mean size dramatically but have only limited effects on the variation of cell size. Furthermore, systems that only act at a single gate for size variation would fail to provide continuous feedback on size variation and would have difficulty correcting noise introduced after that gate operates, which in this case is early in the cell cycle [70]. By contrast, growth rate regulation, particularly sub-exponential growth, where growth rate is proportional to cell mass but exhibits a slope smaller than that of exponential growth, proves to be a highly effective means for reducing cell mass variation throughout the cell cycle (Fig 4 and Section 3 in S1 Text). The effectiveness of this mechanism is bolstered by its operation throughout the entire cell cycle and in the whole cell size range. This form of regulation would be more effective than growth rate modulation restricted to short periods of the cell cycle and only in large cells, as suggested by previous studies [1,8,20,38,49]. Unraveling the determinant factors that underlie the sub-exponential scaling between growth rate and cell mass will likely shed light on the coordination between size-dependent biomass synthesis, nutrient transportation, and macromolecule destruction [71]. We can imagine that pathological conditions, such as aging related diseases, may target growth rate regulation and therefore affect cells at different stages of cell cycle or even non-growing cells.

Aside from the mass-dependent regulation on the G1 length and cell growth rate, the regulation of nonG1 phase lengths also contributes significantly to the reduction of cell mass variation (Fig 5). This is presumably due to the fact that cell cycle phases outside of G1 have non-negligible negative correlations with cell mass (Fig 3) and often occupy a larger portion of the cell cycle than the G1 phase (S2 Table). The mechanisms regulating G2 length have been mainly studied in fission yeast, where the G2/M transition acts as the major size control checkpoint [17,72–74]. Mammalian cells share homologous components of this G2/M regulation with fission yeast [75,76], suggesting that similar mechanisms might function during this stage in mammalian cells. However, further investigation beyond citing simple homology will be needed to confirm this possibility. The regulation of S phase length as a means for controlling cell size in mammalian cells has been rarely explored. One potential mechanism of size-dependent S phase length regulation could involve the control of the number of replication complexes. If the number of forks were proportional to the total cell size, so that small cells made fewer forks, this could serve to lengthen S phase [77]. If cell size were to affect the number of active origins or DNA replication speed, it might also affect the level of DNA damage due to the under-replicated regions [77–79]. Replication stress is not uncommon in normal cycling populations, as evidenced by the presence of DNA lesions in more than 20% of G1 cells in non-transformed cell lines [80]. If the occurrence of replication stress were influenced by cell size and if it led to forms of DNA damage that could be resolved, it could potentially drive tumorigenesis or senescence in a cell size-dependent manner, resulting in heterogeneous behavior in a genetically uniform population. This scenario might hold clinical significance and thus deserves further investigation. Additional research is needed to establish the relationship between the probability of replication stress and cell size during S phase. Furthermore, the actual mechanism of S phase length regulation could be more complicated than the size-dependent replication fork number. The negative correlation between cell mass and S phase length is strengthened in palbociclib-treated RPE-1 cells compared to untreated cells (Fig 5), suggesting more complex crosstalk between the G1 and S phase regulation that cannot be fully explained by the size-dependent replication fork number.

In line with previous research [7], we found that both RPE-1 and HeLa cells exhibit adder-like behaviors (Fig 3I–3J). More specifically, they demonstrate sequential adder behaviors, wherein each phase of their cell cycles can be mathematically expressed as an adder, with the correlation between the masses at the beginning and the end of each phase having a slope close to one (S7 Fig). Our focus in this study is not on their adherence to an adder model. Rather, we emphasize the existence of size control mechanisms across all cell cycle phases. Such regulation could manifest at multiple cell cycle checkpoints by controlling the duration of individual cell cycle phases, operate throughout the cell cycle through continuous monitoring and adjusting the rate of mass accumulation, or more likely, be a combination of both. If the time resolution of the measurements were sufficiently high, we might be able to observe that each fine segment of the cell cycle follows an adder behavior. Such a mechanism would require that a cell continually “knows” how large it is and how large it should be at any point of the cell cycle. How might cells sense their size relative to a changing standard that changes with cell cycle progression? How would such a mechanism respond differently in different cell types, different nutrient conditions, and to pharmacological perturbations? A proposed mechanism of cell size sensing relies on some form of disproportionality of molecular components or signals to cell size. For example, cells might sense size through the sub-scaling of inhibitors or super-scaling of activators to regulate their cell cycle length [6,10,44,81]. Cell mass accumulation requires nutrient provision, transcription, translation, and degradation; any rate-limiting step might serve as a size sensor. It has also been proposed that cells may sense size and modulate growth rate by DNA limitation, cytoplasmic dilution, surface-to-volume ratio, sublinear proportionality between metabolic rate and cell size, transport efficiency, and other such mechanisms [20,70,82–84]. We have found that different cell lines modulate their growth rates differently. It is of course plausible that each cell line we investigated employs a distinct size-sensing mechanism and a distinct mode of response of mass accumulation. However, it seems more likely that all cell lines share a universal mechanism that allows various forms of growth rate modulation under particular conditions. One potential candidate for this universal mechanism would be the mTOR pathway, which governs biomass synthesis and responds to various upstream signals [36,85]. Therefore, we suggest that an investigation of how the mTOR pathway responds to cell size could be informative. Additionally, growth rate regulation exhibits cell cycle-specific patterns and even intrinsic oscillations [29,48,50,51]. The likely coexistence of multiple forms of regulation could complicate any investigation. Future studies might benefit from isolating each mechanism, perhaps by identifying conditions where only one of the processes is dominant. Situations such as cell cycle arrest and size enlargement (so called cellular senescence) triggered by DNA damage or other stresses are of particular interest in this regard [44,86]. Such phenomena may help us disentangle size-dependent growth regulation from other forms of cell cycle-dependent growth regulation, thus allowing us to focus on the effects of cell size on growth rate using the methods we employed in this study.

In summary, the use of ceQPM to quantify single-cell dry mass, mass growth rate, and cell cycle progression has provided the currently most accurate, complete, and quantitative description of cell mass homeostasis in mammalian cells. In this paper, we have also showcased the often-underappreciated power of phenomenological descriptions. Such descriptions have been proven to be inherently powerful in physics and chemistry. The observed reduction in the coefficient variation of cell mass within a proliferating population throughout the cell cycle unequivocally rules out the possibility that cells control mass solely or principally by controlling the length of the G1 phase at the G1/S transition. While this result is far from a complete answer to the problem of cell size homeostasis and does not yet provide specific molecular mechanisms, it nevertheless can serve as a guide for future investigation. It redirects our focus away from the G1/S transition or any specific cell cycle transitions in cell size homeostasis. We propose instead focusing on the molecular-level mechanisms governing size-dependent regulation of growth rate, as this appears to be the predominant player and holds greater promise in elucidating how cells maintain a stable size distribution. Our findings, which reveal compensatory responses to perturbing size, suggest the existence of previously underappreciated regulatory pathways in cell size regulation. Specifically, we suggest that there is a need to examine how cell size feeds back on the anabolic or proteostatic machinery.

As of now, we are still in the early stage of describing the phenomenon of size homeostasis in quantitative terms. These efforts prove that we have much to learn about the regulatory circuits that tell a cell how large it is and how large it should be at any given time or in any given circumstance. Studying cell size homeostasis in cultured cells can lay the groundwork for future investigations into size control in vivo and its implications for disease, thereby expanding our understanding of cell physiology.

## Materials and methods

### Cell culture and chemical treatment

HeLa mAG-hGem, RPE-1 mAG-hGem, HT1080 mAG-hGem mKO2-hCdt1, and HeLa mAG-hGEM DNA-ligase-dsRed cells were made in previous studies by our laboratory [38,87]. U2OS mAG-hGem and Saos-2 mAG-hGEM cells were generated by lentivirus infection in this study. Lentivirus carrying mTurquoise2-SLBP was purchased from Addgene (83842-LV) to make HeLa mAG-hGem mTurquoise2-SLBP, RPE-1 mAG-hGem mTurquoise2-SLBP, and HeLa mAG-hGEM DNA-ligase-dsRed mTurquoise2-SLBP. Single clones of stable expression were selected for each cell line. Cells were incubated at 37°C with 5% CO2 in Dulbecco’s Modified Eagle Medium (DMEM) (11965; Thermo Fisher Scientific) with 25 mM HEPES (15630080; Thermo Fisher Scientific) and 10 mM sodium pyruvate (11360070; Thermo Fisher Scientific), or McCoy’s 5A Medium (16600082; Thermo Fisher Scientific). Both media were supplemented with 10% fetal bovine serum (FBS) (16000044; Thermo Fisher Scientific) and 1% penicillin/streptomycin (15140122; Thermo Fisher Scientific). Palbociclib was purchased from Selleckchem (PD-0332991) and rapamycin was purchased from LC Laboratories (R-5000).

### Live cell imaging

Cells were imaged at 10× magnification by an Eclipse Ti microscope with the Perfect Focus System (PFS) (Nikon, Japan) and an SID4BIO camera (Phasics, France). Nikon NIS-Elements AR ver. 4.13.0.1 software with the WellPlate plugin was used to acquire images. A home-made incubation chamber was used to maintain a constant environment of 36°C and 5% CO2 during imaging. Cells were seeded on 6-well glass bottom plates (P06G-1.5-14-F; MatTek) at a density of 1,500 cells/cm^2^ 3 h before long-term imaging or 3,500 cells/cm^2^ 16 h before short-term imaging. Before time-lapse imaging was started, mineral oil (M8410; Millipore Sigma) was added into each well to prevent media evaporation. In the long-term experiments studying the cell cycle regulation, cells were monitored for 48 or 72 h. In the short-term experiments studying growth rate modulation, cells were monitored for 3 h. For all experiments, the phase images were acquired every 30 min, and the fluorescence images were acquired every 1 h.

### Cell fixation and cell cycle identification

After the short-term time-lapse imaging, the mineral oil was gently removed by aspiration. Cells were fixed with 4% paraformaldehyde (RT 157–8; Electron Microscopy Sciences) and stained with Hoechst 33342 (62249; Thermo Fisher Scientific) at a final concentration of 1 μm. The cells were then imaged by QPM again to identify their cell cycle stages.

### QPM image processing and data analysis

The QPM images were processed by the ceQPM method developed previously [29] and conducted on the O2 high-performance computing cluster at Harvard Medical School.

To test the significance of the minimal cell cycle phase length, we fitted the binned correlations between the initial mass and cell cycle phase duration in Figs 3 and 5 with 2 alternative models. A linear model *y* = *a*_1_*x*+*b*_1_, and a bilinear model $y=a_{2}x+b_{2}(x\leq x_{0}),y=a_{2}x_{0}+b_{2}(x>x_{0})$, where *y* is the cell cycle phase length, *x* is the initial mass, *a*_1_, *b*_1_, *a*_2_, *b*_2_, and *x*_0_ are the fitting parameters. We used the Akaike information criterion (AIC) to compare the goodness of fits. A smaller AIC indicates a better fit, and the relative likelihood p_linear or p_bilinear predicts the probability that the alternative model is a better fit when the linear or bilinear model has the smaller AIC [88]. Since the correlations between the initial mass and cell cycle phase duration were not linear, we utilized the Kendall’s rank correlation coefficient to represent the correlation strength. This coefficient is more suitable for our data as it does not assume a linear relationship, unlike the widely used Pearson correlation coefficient [89].

To evaluate whether the cell cycle control could explain the adder behavior in Fig 3I and 3J, we assumed cells grow exponentially at the rate of *α* = ln(2)/*DT*, where *DT* is the averaged cell cycle length. The division mass could be predicted by *m*_*d*_ = *m*_*b*_*e*^*αT*^, *T* = *f*(*m*_*b*_), where *f* is the best-fitted function in the alternative models of cell cycle length versus birth mass.

To fit the binned correlation between growth rate and cell mass in Figs 4 and 5, we employed 2 alternative models: a linear model $\frac{dm}{dt}=\alpha m+\beta$ and a bilinear model $\frac{dm}{dt}=(am+b)(m<m_{\tau })+(\gamma m+am_{\tau }+b-\gamma m_{\tau })(m\geq m_{\tau })$, where *y* is the growth rate, *x* is the cell mass, *α*, *β*, *a*, *b*, *γ*, and *m*_*τ*_ are the fitting parameters. We used the AIC to estimate the goodnesses of fits.

## Supporting information

S1 Text Models used in this study. (DOCX)

S1 FigThe left- and right-hand sides of Eq 1 and their difference quantified in HeLa cells.In the term, ${CV}_{d}^{2}+Q^{2},{CV}_{d}^{2}$ is indicated in black, *Q*^2^ is indicated in white; error bars are the standard deviation of 8 experiments. The data underlying this figure and the scripts used to generate the plots are available on the Open Science Framework at osf.io/3kyvw.(EPS)

S2 Fig RPE-1 and U2OS sensitivity to palbociclib. The mean cell mass of the population (A) and the percentage of G1 cells quantified by low Geminin expression (B) after being treated in palbociclib at the indicated concentrations for 2 days. Dashed black lines show the concentration (50 nM) chosen for the analyses in this study. The data underlying this figure and the scripts used to generate the plots are available on the Open Science Framework at osf.io/3kyvw.
(EPS)

S3 Fig **mAG-hGeminin (A) and cell mass (B) trajectories of a representative HeLa cell.** Dashed lines denote the timing of the G1/S transition identified by the initiation of geminin accumulation. The data underlying this figure and the scripts used to generate the plots are available on the Open Science Framework at osf.io/3kyvw.
(EPS)

S4 Fig Segregation of cells into stages along the cell cycle mean path. (A) The 2D plane of the logarithmic scale of mAG-hGeminin intensity, log(Geminin), and the intensity of Hoechst fluorescence, DNA, in asynchronous RPE-1 cells. Black contours indicate cell number density; the solid red line is the cell cycle mean path; filled red circles show the centroids of the chosen stages along the mean path; the stages are evenly separated in the time axis computed by the ERA method [38]. (B–E) The averages of log(Geminin) (blue) and DNA content (red) change with cell cycle progression in different cell lines. X-axes are calculated by the ERA method [38]. The cell cycle is segregated into 4 phases indicated by color-shaded areas: the early G1 phase from birth to the onset of geminin accumulation, the late G1 phase from the initiation of geminin accumulation to the onset of DNA replication, the S phase covering DNA replication, and the G2-M phase where geminin and DNA accumulation plateau. (F) Error in computed cell mass CV caused by inaccurate cell cycle stage identification. The cell dry mass and cell cycle markers data were from Fig 2D. We added 10% random Gaussian noise to each cell’s position in the log(Geminin)-DNA plane. The cells were reassigned to cell cycle stages according to their new positions, and the cell mass CV of each stage was computed. The solid black line and error bars indicate the mean and standard deviation of computed cell mass CVs of 100 simulations; the first and last stages were truncated due to having much higher cell numbers and variations than other stages. (G, I) The 2D planes of log(Geminin) and DNA content in RPE-1 cells in 50 nM palbociclib (G) or 100 nM rapamycin (I). The red line and filled circles are the cell cycle mean path and centroids of stages calculated from the treated cells. (H, J) The averages of log(Geminin) (blue) and DNA content (red) change with cell cycle progression in RPE-1 cells in 50 nM palbociclib (H) or 100 nM rapamycin (J). The data underlying this figure and the scripts used to generate the plots are available on the Open Science Framework at osf.io/3kyvw.
(EPS)

S5 Fig The geminin and SLBP markers faithfully report the timing and duration of S phase. (A) The trajectories of dsRed-DNA-ligase I foci, mAG-hGeminin, and mTurquoise2-SLBP in a representative HeLa cell. Open circles are the raw data; solid colored lines are the spline interpolations; dashed yellow and pink lines mark the S phase start and end, respectively. (B–D) Correlations between the S phase start (B), end (C), and duration (D) identified by the dsRed-DNA-ligase foci or mAG-hGeminin and mTurquoise2-SLBP combined. Each black dot is one observation; Solid red lines are the best linear fit. Texts indicate the functions of the solid red lines and the Pearson correlations of the black dots. The data underlying this figure and the scripts used to generate the plots are available on the Open Science Framework at osf.io/3kyvw.
(EPS)

S6 Fig **The impact of minimal cell cycle length on cell mass homeostasis, indicated by the birth mass CV (A) and mean birth mass (B) changing with simulated generations.** Different colors show the percentage of cells affected by the minimal cell cycle length in the population of the first generation of simulations. The data underlying this figure and the scripts used to generate the plots are available on the Open Science Framework at osf.io/3kyvw.
(EPS)

S7 Fig The sequential adder behavior in RPE-1 and HeLa cells. (A, D) The correlations between birth mass and mass at G1/S in RPE-1 (A) and HeLa (D) cells. (B, E) The correlations between mass at G1/S and mass at S/G2 in RPE-1 (B) and HeLa (E) cells. (C, F) The correlations between mass at S/G2 and division mass in RPE-1 (C) and HeLa (F) cells. Each gray dot is an observation; black squares are the average of each cell mass bin; error bars are the standard error of means (SEMs). Solid black lines are the best linear fits of the gray dots; texts indicate the functions of the solid black lines. The data underlying this figure and the scripts used to generate the plots are available on the Open Science Framework at osf.io/3kyvw.
(EPS)

S8 Fig Growth rate modulation in HeLa cells. (A) The correlation between cell mass and growth rate in HeLa cells when pooling all cells together. Each gray dot is an observation in the 3-h measurements, *n* = 18,334. Black squares are the median growth rate of each mass bin; error bars are SEMs. The solid black line is the best fit of the black squares (S5 Table). The dashed black line indicates exponential growth. (B, C) The correlations between cell mass and growth rate in HeLa cells in 4 cell cycle phases (B) and one fine stage of the cell cycle (C). The stages were determined by log(Geminin) and DNA using the ERA method [38], as indicated in S4C Fig. Filled squares are the median growth rate of each mass bin; error bars are SEMs. The solid lines are the best fit of the filled squares (S5 Table). The dashed black line in (B) indicates exponential growth. (D) The slope of the linear relationship between cell mass and growth rate plotted against cell cycle progression. The short-dashed line indicates the expected slope for exponential growth. The data underlying this figure and the scripts used to generate the plots are available on the Open Science Framework at osf.io/3kyvw.
(EPS)

S9 Fig **Specific growth rate changes with cell cycle progression in RPE-1 (A) and HeLa cells (B) in G1 (blue) and nonG1 (red) phases.** Since the binned correlation could be affected by inspection bias [52], we investigated how the specific growth rate (growth rate divided by mass) changed with cell cycle progression from the long-term trajectories as recommended by Kar and colleagues [52]. We arbitrarily assumed the G1 or nonG1 phase each occupies half of the cell cycle when normalizing the length of the growth trajectories. Solid blue and red lines are the means of the normalized growth trajectories of the G1 and nonG1 segments; the shaded areas indicate SEM. Dashed lines are the expected curves of exponential growth; short-dashed lines are the expected curves of linear growth, assuming the cells behave like an adder. The data underlying this figure and the scripts used to generate the plots are available on the Open Science Framework at osf.io/3kyvw.
(EPS)

S10 Fig Simulation results for the sub-exponential growth rate modulation. (A, C, F) Contour plots illustrating the rate of change in cell mass CV at the beginning of the cell cycle (*t*′ = 0) when assuming *CV*_*α*′_ = *CV*_*β*′_ (A), *CV*_*β*′_ = 0 (C), or *CV*_*α*′_ = 0 (F), respectively. Here, *μ*_*α*′_ represents the mean of *α*′. (B, D, G) Contour plots illustrating the rate of change in cell mass CV at the end of the cell cycle (*t*′ = 1) when assuming *CV*_*α*′_ = *CV*_*β*′_ (B), *CV*_*β*′_ = 0 (D), or *CV*_*α*′_ = 0 (G), respectively. (E, H) Contour plots illustrating the overall change in cell mass CV throughout the cell cycle when assuming *CV*_*β*′_ = 0 (E), or *CV*_*α*′_ = 0 (H), respectively. Solid circles indicate the corresponding positions in the contour plots when adopting parameter values from the experimental observations of RPE-1 and HeLa cells. The data underlying this figure and the scripts used to generate the plots are available on the Open Science Framework at osf.io/3kyvw.
(EPS)

S11 FigEstimating the variability in *α*′ for RPE-1 and HeLa cells.(A, B) The variability of the specific growth rate, defined as the growth rate divided by cell mass, does not change with cell mass for RPE-1 (A) and HeLa (B) cells. Blue squares and lines indicate the means and standard deviations of live cell growth trajectories, which are binned by cell mass. The black lines show $(1\pm \bar{CV}){\bar{gr}}_{j}$, where $\bar{CV}$ is the average CV in specific growth rate for all cell mass bins, and ${\overset{-}{gr}}_{j}$ is the average specific growth rate for each bin. (C) Schematic illustrating the definitions of intercellular and intracellular variability in *α*′. Solid lines are representative live cell growth trajectories. Dashes lines represent the means of each trajectory. Intercellular variabilty is defined as the variaiton among the means of each trajectories, while intracellular variability is defined as the fluctruation within individual trajectories. The data underlying this figure and the scripts used to generate the plots are available on the Open Science Framework at osf.io/3kyvw.(EPS)

S12 FigSimulation results for the Bilinear growth rate modulation.(A–D) Three-dimensional (3D) volumetric plots showing how the change in cell mass CV throughout the cell cycle responds to the means of *γ*′ and $m_{\tau }^{\prime }$, represented by *μ*_*α*′_ and $\mu_{m_{\tau }^{\prime }}$, when applying variability (CV) to only 1 parameter of *α*′ (A), *γ*′ (B), $m_{\tau }^{\prime }$ (C), or equal variability to all 3 parameters (D). The slice planes are orthogonal to the CV axis at CV = 0.2. (E, F) Contour plots illustrating the change in cell mass CV during the G1 (E) and nonG1 phases (F) with the means of *γ*′ and $m_{\tau }^{\prime }$ when assuming a 30% CV in *α*′. (G, H) Contour plots illustrating the change in cell mass CV during the G1 (G) and nonG1 phases (H) with means of *γ*′ and $m_{\tau }^{\prime }$ when assuming a 40% CV in *α*′. Solid circles in (E–H) indicate the corresponding positions in the contour plots when adopting parameter values from the experimental data. The data underlying this figure and the scripts used to generate the plots are available on the Open Science Framework at osf.io/3kyvw.(EPS)

S13 Fig Impact of growth rate variability on division mass CV, *CV*(*m*_*i*_(*T*_*i*_)), in the stochastic model. The stochastic model is described in Section 4, Scenario IX in S1 Text. All parameter values used in this simulation are listed in the table at the end of Section 4, with the exception of *CV*_*gr*_, which is varied in this simulation. Solid blue lines indicate the simulation results. Filled blue circles are the division mass CV when simulated with the *CV*_*gr*_ estimated from experimental data. Dashed black lines represent the division mass CV measured in experiments. The data underlying this figure and the scripts used to generate the plots are available on the Open Science Framework at osf.io/3kyvw.
(EPS)

S1 Table Characteristics of the human cell lines used in this study. (DOCX)

S2 Table The durations of cell cycle phases for HeLa, RPE-1, RPE-1 in 100 nM rapamycin or 50 nM palbociclib at cell mass homeostasis. MAD is the median absolute deviation, and nMAD is MAD normalized by the median in robust statistics.
(DOCX)

S3 Table Comparing cell cycle phase durations and mass versus phase length correlations with and without the mTurquoise2-SLBP marker in HeLa cells. (DOCX)

S4 Table Comparison of the linear and bilinear fits for the cell mass vs. cell cycle phase length correlations. The significantly better fits (p_bilinear or p_linear < 0.05) and the significant negative correlations (*p* < 0.05) are highlighted.
(DOCX)

S5 Table Comparison of the linear and bilinear fits for the cell mass vs. growth rate correlations. The significantly better fits (p_bilinear or p_linear < 0.05) are highlighted.
(DOCX)

S6 TableThe normalized fitting parameters for the cell mass vs. growth rate correlations for different cell lines.For correlations fitted better by the linear model, $\frac{dm}{dt}=\alpha m+\beta$, the normalized parameters *α*′ and *β*′ are listed in the table, with *α*′ = *α*<*T*>, $\beta^{\prime }=\beta \frac{<T>}{<m_{b}>}$, where <*T*> and <*m*_*b*_> are the means of cell cycle length and cell birth mass, respectively. For exponential growth, *α*′ = *ln*2 ~= 0.693. For correlations fitted better by the bilinear model, $\frac{dm}{dt}=(am+b)(m<m_{\tau })+(\gamma m+am_{\tau }+b-\gamma m_{\tau })(m\geq m_{\tau })$, the normalized parameters *a*′, *b*′, *γ*′, and $m_{\tau }^{\prime }$ are listed in the table, with *a*′ = *a*<*T*>, $b^{\prime }=b\frac{<T>}{<m_{b}>},\gamma^{\prime }=\gamma <T>,m_{\tau }^{\prime }=\frac{m_{\tau }}{<m_{b}>}$. The correlation slopes, *α*′, *a*′, and *γ*′, lower than 0.75 or higher than 1.25-fold (arbitrarily chosen thresholds) of *ln*2 were highlighted. SE and BI denote the type of growth rate modulation, where SE stands for sub-exponential and BI stands for bilinear.(DOCX)

S7 Table The values of *λ*′ and *α*′ used in Fig 5L, for untreated HeLa and RPE-1 cells, as well as RPE-1 cells treated with 50 nM palbociclib or 100 nM rapamycin. (DOCX)

S8 Table Contribution of each factor to cell mass variation, as indicated by the division mass CV simulated using the stochastic model in Section 4 in S1 Text. The values reported in this table are the average of division mass CVs obtained from 50 simulations.
(DOCX)

S9 Table The frequencies of cell death, cell cycle arrest, and cytoplasmic loss observed in the long-term measurements in HeLa, RPE-1, RPE-1 in 100 nM rapamycin, and RPE-1 in 50 nM palbociclib when cells have reached cell mass homeostasis. (DOCX)

S10 Table Birth size CVs, division size CVs, and DA stds. reported in the literature. (DOCX)

S1 Movie Time-lapse quantitative phase images of RPE-1 cells in 50 nM palbociclib; the time interval is 30 min; the yellow arrow indicates the lost cytoplasmic mass of a mitotic cell (red arrow); the scale bar indicates 100 μm. (GIF)

## Abbreviations

AIC: Akaike information criterion
BI: bilinear
ceQPM: computationally enhanced quantitative phase microscopy
CV: coefficient of variation
DA std: standard deviation of Division Asymmetry
DMEM: Dulbecco’s Modified Eagle Medium
ERA: ergodic rate analysis
FBS: fetal bovine serum
PFS: Perfect Focus System
Rb: retinoblastoma
SE: sub-exponential
SLBP: stem-loop binding protein
