# Supplementary material for: Cell size homeostasis is tightly controlled throughout the cell cycle
Source: PLoS Biol. 2024 Jan 5;22(1):e3002453. doi: 10.1371/journal.pbio.3002453 (PMC10769027; doi:10.1371/journal.pbio.3002453)
Supplement: S1 Text — (DOCX) [file pbio.3002453.s001.docx]

**S1 Text. Models used in this study**

Table of Contents

1. An abstract model of cell mass homeostasis 2
2. Simulation of the impact of minimal cell cycle length on cell mass variation 4
3. The impact of different forms of growth modulation on cell mass variation5
   1. Sub-exponential growth rate modulation 5

3.1.1 Analytic solutions5

3.1.2 Numerical simulations8

- 1. Bilinear growth rate modulation 10

1. Simulations of the contribution of each control mechanism on cell mass variation in untreated HeLa and RPE-1 cells, as well as RPE-1 cells treated with palbociclib or rapamycin11
2. An abstract model of cell mass homeostasis

In this section, we outline the abstract model employed to simulate the results in Fig 1A-C. The purpose of developing this model is to investigate the effects of different G1 regulation strengths, which we characterize as the slope of the correlation between birth mass and G1 length. In this stochastic model, we make the following assumptions: all cells accumulate mass exponentially, the length of the nonG1 phase remains constant, and the G1 length regulation is the only means to control cell mass.

The G1 duration is determined by birth mass:

$G1=\left( a_{G1}m_{b}+b_{G1} \right)\left( m_{b}\leq{x0}_{length\_G1} \right)+\left( a_{G1}{x0}_{length\_G1}+b_{G1} \right)\left( m_{b}>{x0}_{length\_G1} \right)$,

where $m_{b}$ denotes birth mass, $a_{G1}$, $b_{G1}$, and ${x0}_{length\_G1}$ are parameters reflecting the strength of the G1 length regulation. By varying these parameters, we simulate the behaviors of cell populations with different G1 regulation strengths.

For simplicity, we introduced Gaussian noise to the division mass at the initiation of each simulation, the cell cycle length, and the exponent of exponential growth, with their means and coefficients of variation (CVs) summarized in the Table in this section. In each iteration, we simulated a population of 1000 cells for 20 generations.

At the end of each generation, cells divide their mass in half with Gaussian noise, $d_{CV}$, to represent asymmetrical partition. Only one of the two daughter cells is retained for the subsequent generation. We also implemented a cutoff for the entire cell cycle length, $T_{max}$. If the sum of the G1 and nonG1 lengths of a cell exceeds $T_{max}$, its cell cycle length is truncated at $T_{max}$.

The cell mass of cell $i$ in generation $k$ at the time step $j$, $m_{j}^{i,k}$, is determined by

$m_{j}^{i,k}=m_{j-1}^{i,k}+m_{j-1}^{i,k}\alpha^{i,k}dt$, where $j=2,\ldots,[\frac{T^{i,k}}{dt}]$.

where $\alpha^{i,k}$and $T^{i,k}$ are the growth exponent and the cell cycle length of cell $i$ in generation $k$, respectively.

The simulation results are presented in Fig 1A-C.

**Table. The meanings and values of parameters used to simulate the results in Fig 1A-C.**

| **Parameter** | **Meaning** | **Blue**  **No G1 length regulation** | **Red**  **Strong G1 length regulation** | **Yellow**  **Weak G1 length regulation** |
| --- | --- | --- | --- | --- |
| $\mu_{md}$ | The mean cell mass at division within the population at the initiation of each simulation | 1000 | - | - |
| $CV_{md}$ | The CV of the cell mass at division within the population at the initiation of each simulation | 0.15 | - | - |
| $d_{CV}$ | The CV of the partition error | 0.05 | - | - |
| $T_{max}$ | The maximum cell cycle length | 48 | - | - |
| $\mu_{nonG1}$ | The mean nonG1 length | 12.5 | - | - |
| $CV_{T}$ | The CV of the cell cycle length | 0.18 | - | - |
| $dt$ | The time step of simulations | 0.5 | - | - |
| $\mu_{\alpha}$ | The mean growth exponent | 0.0277 |  |  |
| ${CV}_{\alpha}$ | The CV of the growth exponent | 0.15 | - | - |
| $a_{G1}$ | Parameter of the G1 regulation | 0 | -0.053 | -0.017 |
| $b_{G1}$ | Parameter of the G1 regulation | 12.5 | 39 | 21 |
| ${x0}_{length\_G1}$ | Parameter of the G1 regulation | Inf | 717 | 1176.5 |

1. Simulation of the impact of minimal cell cycle length on cell mass variation

In this section, we describe the stochastic model we used for generating the results in S6 Fig.

To investigate the impact of minimal cell cycle length on cell mass variation, we simulated cell populations across multiple generations, assuming exponential growth in individual cell masses. The birth mass of cells in the first generation, ${mb}_{i}^{1}$, follows a normal distribution with a mean of $\mu_{mb}$ and a standard deviation of $\mu_{mb}*CV_{mb}$. Here, $i=1,\ldots,10000$ is the ID of the cells.

${mb}_{i}^{1}\sim N(\mu_{mb},{(\mu_{mb}*CV_{mb})}^{2})$ .

The cell cycle length of cell $i$ in generation $j$, $T_{i}^{j}$, is determined by the birth mass, ${mb}_{i}^{j}$, according to a bilinear function:

$T_{i}^{j}=(\left( a\cdot{mb}_{i}^{j}+b \right)\left( {mb}_{i}^{j}\leq m_{thresh} \right)+\left( a\cdot m_{thresh}+b \right)\left( {mb}_{i}^{j}>m_{thresh} \right))(1+\delta_{t})$,

where $m_{thresh}$ represents the 100^th^, 90^th^, 80^th^, 70^th^, 60^th^, or 50^th^ percentile of the ${mb}_{i}^{1}$ distribution. $\delta_{t}$ accounts for noise in the cell cycle length, which follows a normal distribution, $\delta_{t}\sim N(0,{CV}_{t}^{2})$.

The cell mass at division, ${md}_{i}^{j}$, is determined by

${md}_{i}^{j}={mb}_{i}^{j}e^{\alpha T_{i}^{j}}$,

where $\alpha$ represents the exponent of exponential growth.

The birth mass of the subsequent generation is determined by

$${mb}_{i}^{j+1}={md}_{i}^{j}\left( 0.5+\delta_{p} \right),$$

where $\delta_{p}$ represents the noise in the symmetric partition and follows a normal distribution, $\delta_{p}\sim N(0,{p\_err}^{2})$.

The simulation was conducted for 100 generations.

**Table. Parameter values used for generating results in S6 Fig.**

| $\mu_{mb}$ | 484 |
| --- | --- |
| $CV_{mb}$ | 0.18 |
| $p\_err$ | 0.051 |
| $\alpha$ | 0.0248 |
| $CV_{t}$ | 0.15 |
| $a$ | -0.0213 |
| $b$ | 38.3 |

The parameter values of $\mu_{mb}$, $CV_{mb}$, $\alpha$, $CV_{t}$, $a$, $b$, and $p\_err$ were derived from the HeLa cell data.

1. The impact of different forms of growth modulation on cell mass variation

In this section, we examined the impact of growth rate modulation and growth rate noise on the coefficient of variation in cell mass over a single cell cycle. We assumed that all cells divided at the same cell cycle length and that all populations started with the same variation in birth mass. To address this problem, we utilized a combination of analytical solutions and numerical simulations.

**3.1 Sub-exponential growth rate modulation**

When growth rate modulation is in the Sub-exponential (SE) form, the correlation between cell mass and growth rate can be expressed as

$\frac{dm}{dt}=\alpha m+\beta$. (Eq. 2)

Equation (2) has the following solution with initial condition $m(0)=m_{0}$:

$m(t)=m_{0}e^{\alpha t}+\beta\left( e^{\alpha t}-1 \right)/\alpha$. (Eq. 3)

Here, $m(t)$, $m_{0}$, $\alpha$, $\beta$, and $t$ are normalized by the mean birth mass and the mean cell cycle length.

**3.1.1 Analytical solutions**

We are interested in understanding how the coefficient of variation (CV) of $m(t)$ changes, particularly in identifying the parameters that lead to a decrease in CV over time.

When $\beta=0$, cell mass accumulates exponentially.

We have

${CV}_{m(t)}^{2}=\frac{\langle{m(t)}^{2}\rangle-{\langle m(t)\rangle}^{2}}{{\langle m(t)\rangle}^{2}}$*.*

The variability in $\alpha$ arises from two sources: the stochastic partitioning of cellular contents during cell division and intrinsic fluctuations in biochemical reactions (1). For the sake of mathematical simplicity, we assume that the mean and CV of $\alpha$ do not change with time and are independent of $m$. The assumption that CV of $\alpha$ is independent of cell mass is supported by the experimental data in S11A-B Fig. Both $m_{b}$ and $\alpha$ follow normal distributions. Then, by using the property of moment-generating function, the CV of $m(t)$, ${CV}_{m(t)}^{2}$, can be calculated analytically as:

${CV}_{m(t)}^{2}=\frac{\langle m_{0}^{2}\rangle\langle e^{2\alpha t}\rangle-{\langle m_{0}\rangle}^{2}{\langle e^{\alpha t}\rangle}^{2}}{{\langle m_{0}\rangle}^{2}{\langle e^{\alpha t}\rangle}^{2}}=\frac{(\sigma_{m}^{2}+\mu_{m}^{2})e^{2\mu_{\alpha}t+2\sigma_{\alpha}^{2}t^{2}}-\mu_{m}^{2}e^{2\mu_{\alpha}t+\sigma_{\alpha}^{2}t^{2}}}{\mu_{m}^{2}e^{2\mu_{\alpha}t+\sigma_{\alpha}^{2}t^{2}}}$,

${CV}_{m(t)}^{2}=\left( {CV}_{m_{0}}^{2}+1 \right)e^{\sigma_{\alpha}^{2}t^{2}}-1$ ,

which shows that the CV of $m(t)$ increases super-exponentially with cell cycle progression if there were no control mechanisms.

When $\beta\neq0$, the CV of $m(t)$ becomes too complex to obtain a closed-form solution, but we can still apply a Taylor expansion to the solution to determine how CV changes at the beginning of the cell cycle:

$e^{\alpha t}=\sum_{n=0}^{\infty} \frac{\left( \alpha t \right)^{n}}{n!}$, and $\left( e^{\alpha t}-1 \right)/\alpha=t\sum_{n=0}^{\infty} \frac{\left( \alpha t \right)^{n}}{(n+1)!}$.

Generically, we have:

$$\langle{m(t)}^{2}\rangle=\langle m_{0}^{2}\rangle\langle e^{2\alpha t}\rangle+2\langle m_{0}\rangle\langle\beta e^{\alpha t}\left( e^{\alpha t}-1 \right)/\alpha\rangle+{\langle m_{0}\rangle}^{2}\langle\beta^{2}\left( e^{\alpha t}-1 \right)^{2}/\alpha^{2}\rangle$$

$=\langle m_{0}^{2}\rangle\langle e^{2\alpha t}\rangle+2t\sum_{n,m=0}^{\infty} \frac{{\langle\alpha}^{n+m}\beta\rangle}{n!(m+1)!}t^{n+m}+t^{2}\sum_{n,m=0}^{\infty} \frac{{\langle\alpha}^{n+m}\beta^{2}\rangle}{(n+1)!(m+1)!}t^{n+m}$,

$${\langle m(t)\rangle}^{2}={\langle m_{0}\rangle}^{2}{\langle e^{\alpha t}\rangle}^{2}+2\langle m_{0}\rangle\langle e^{\alpha t}\rangle\langle\beta\left( e^{\alpha t}-1 \right)/\alpha+{\langle m_{0}\rangle}^{2}{\langle\beta\left( e^{\alpha t}-1 \right)/\alpha\rangle}^{2}$$

$={\langle e^{\alpha t}\rangle}^{2}+2t\sum_{n,m=0}^{\infty} \frac{{\langle\alpha}^{n}\rangle\langle\alpha^{m}\beta\rangle}{n!(m+1)!}t^{n+m}+t^{2}\sum_{n,m=0}^{\infty} \frac{{\langle\alpha}^{n}\beta\rangle\langle\alpha^{m}\beta\rangle}{(n+1)!(m+1)!}t^{n+m}$,

where $\langle m_{0}\rangle=1$ due to the normalization.

Therefore if we only keep lower-order terms, we have:

$\langle{m(t)}^{2}\rangle\approx\left( \sigma_{m}^{2}+1 \right)+2\left[ \left( \sigma_{m}^{2}+1 \right)\langle\alpha\rangle+\langle\beta\rangle\right]+\left[ 2(\sigma_{m}^{2}+1)\langle\alpha^{2}\rangle+3\langle\alpha\beta\rangle+\langle\beta^{2}\rangle\right]t^{2}$,

$${\langle m\left( t \right)\rangle}^{2}\approx1+2\left[ \langle\alpha\rangle+\langle\beta\rangle\right]t$$

$+\left[ \left( \langle\alpha^{2}\rangle+{\langle\alpha\rangle}^{2} \right)+\left( \langle\alpha\beta\rangle+2\langle\alpha\rangle\langle\beta\rangle\right)+{\langle\beta\rangle}^{2} \right]t^{2}$.

So the derivatives are approximately:

$\frac{d}{dt}\langle{m(t)}^{2}\rangle\approx2\left[ (\sigma_{m}^{2}+1)\langle\alpha\rangle+\langle\beta\rangle\right]+2\left[ 2(\sigma_{m}^{2}+1)\langle\alpha^{2}\rangle+3\langle\alpha\beta\rangle+\langle\beta^{2}\rangle\right]t$,

$${\frac{d}{dt}\langle m\left( t \right)\rangle}^{2}\approx2\left[ \langle\alpha\rangle+\langle\beta\rangle\right]$$

$+2\left[ \left( \langle\alpha^{2}\rangle+{\langle\alpha\rangle}^{2} \right)+\left( \langle\alpha\beta\rangle+2\langle\alpha\rangle\langle\beta\rangle\right)+{\langle\beta\rangle}^{2} \right]t$.

Since we are mostly interested in whether the CV is increasing or decreasing, i.e., the sign of $d_{t}CV$, we only need to compute the numerator part of the derivative. Combining, we have:

$$\frac{{dCV}_{m(t)}^{2}}{dt}\propto\frac{d\langle{m(t)}^{2}\rangle}{dt}{\langle m(t)\rangle}^{2}-\langle{m(t)}^{2}\rangle\frac{d{\langle m(t)\rangle}^{2}}{dt}$$

$$\approx2\left[ (\sigma_{m}^{2}+1)\langle\alpha\rangle+\langle\beta\rangle\right]-2(\sigma_{m}^{2}+1)\left[ \langle\alpha\rangle+\langle\beta\rangle\right]$$

$$+4\left[ (\sigma_{m}^{2}+1)\langle\alpha\rangle+\langle\beta\rangle\right]\left[ \langle\alpha\rangle+\langle\beta\rangle\right]t-4\left[ \langle\alpha\rangle+\langle\beta\rangle\right]\left[ (\sigma_{m}^{2}+1)\langle\alpha\rangle+\langle\beta\rangle\right]t$$

$$+2\left[ 2\left( \sigma_{m}^{2}+1 \right)\langle\alpha^{2}\rangle+3\langle\alpha\beta\rangle+\langle\beta^{2}\rangle\right]t$$

$$-2(\sigma_{m}^{2}+1)\left[ \left( \langle\alpha^{2}\rangle+{\langle\alpha\rangle}^{2} \right)+\left( \langle\alpha\beta\rangle+2\langle\alpha\rangle\langle\beta\rangle\right)+{\langle\beta\rangle}^{2} \right]t$$

$\approx-2\sigma_{m}^{2}\mu_{\beta}+2\left[ (\sigma_{m}^{2}+1)\sigma_{\alpha}^{2}+\sigma_{\beta}^{2}+(2-\sigma_{m}^{2})\sigma_{\alpha\beta}-\sigma_{m}^{2}{\mu_{\beta}}^{2}-3\sigma_{m}^{2}\mu_{\alpha}\mu_{\beta} \right]t$.

Given our assumption of $\mu_{\beta}\geq0$, the 0th order is always non-positive. Thus, when $\beta\neq0$, the cell mass CV always decreases at the beginning of the cell cycle. Additionally, the linear term suggests that if $\beta$ is not large enough, the cell mass CV could eventually increase after an initial brief decline.

The results above show that the cell mass variation consistently decreases to a certain extent at the beginning of the cell cycle. However, they do not indicate whether the overall cell mass variation can be maintained or reduced after an entire cell cycle ($t=1$):

${CV}_{m(1)}\leq{CV}_{m(0)}$.

Because of the nature of Taylor expansion, the results above cannot be readily extended to the scenario where $t=1$. Therefore, rather than presenting an exact analytical solution, we will now derive a lower bound for $\mu_{\beta}$ that leads to a reduction in cell mass variation.

First, we notice that ${CV}_{m(1)}\leq{CV}_{m(0)}$ can be rephrased as determining the condition under which the following inequality holds:

$\frac{\langle{m(1)}^{2}\rangle-{\langle m(1)\rangle}^{2}}{{\langle m(1)\rangle}^{2}}\leq\frac{\langle m_{0}^{2}\rangle-{\langle m_{0}\rangle}^{2}}{{\langle m_{0}\rangle}^{2}}$.

Since we also require that cells can maintain the same division mass, we have the condition:

$\langle m(1)\rangle=2\langle m_{0}\rangle=2$.

The inequality above simply becomes:

$\langle{m(1)}^{2}\rangle\leq4\left( \sigma_{m}^{2}+1 \right)$.

From above we find that

$$\langle{m(1)}^{2}\rangle=\langle m_{0}^{2}\rangle\langle e^{2\alpha}\rangle+2\sum_{n,m=0}^{\infty} \frac{{\langle\alpha}^{n+m}\beta\rangle}{n!(m+1)!}+\sum_{n,m=0}^{\infty} \frac{{\langle\alpha}^{n+m}\beta^{2}\rangle}{(n+1)!(m+1)!}<\langle m_{0}^{2}\rangle\langle e^{2\alpha}\rangle+2\sum_{n,m=0}^{\infty} \frac{{\langle\alpha}^{n+m}\beta\rangle}{n!m!}+\sum_{n,m=0}^{\infty} \frac{{\langle\alpha}^{n+m}\beta^{2}\rangle}{n!m!}$$

$=\left( \sigma_{m}^{2}+1 \right)\langle e^{2\alpha}\rangle+2\langle\beta e^{2\alpha}\rangle+\langle\beta^{2}e^{2\alpha}\rangle$,

$$\langle\beta e^{2\alpha}\rangle=\langle\beta e^{2\sigma_{\alpha\beta}\frac{\beta-\mu_{\beta}}{\sigma_{\beta}^{2}}}\rangle e^{2\mu_{\alpha}+2\sigma_{\alpha}^{2}\left( 1-\rho^{2} \right)}=\left( \mu_{\beta}+2\sigma_{\alpha\beta} \right)e^{2\sigma_{\alpha}^{2}\rho^{2}+2\mu_{\alpha}+2\sigma_{\alpha}^{2}\left( 1-\rho^{2} \right)}$$

$=\left( \mu_{\beta}+2\sigma_{\alpha\beta} \right)e^{2\left( \mu_{\alpha}+\sigma_{\alpha}^{2} \right)}$,

$\langle\beta^{2}e^{2\alpha}\rangle=\langle\beta^{2}e^{2\sigma_{\alpha\beta}\frac{\beta-\mu_{\beta}}{\sigma_{\beta}^{2}}}\rangle e^{2\mu_{\alpha}+2\sigma_{\alpha}^{2}\left( 1-\rho^{2} \right)}=\left[ {\sigma_{\beta}^{2}+\left( \mu_{\beta}+2\sigma_{\alpha\beta} \right)}^{2} \right]e^{2\left( \mu_{\alpha}+\sigma_{\alpha}^{2} \right)}$,

$\langle{m\left( 1 \right)}^{2}\rangle<\left[ \sigma_{m}^{2}+1+2\left( \mu_{\beta}+2\sigma_{\alpha\beta} \right){+\left( \mu_{\beta}+2\sigma_{\alpha\beta} \right)}^{2}+\sigma_{\beta}^{2} \right]e^{2\left( \mu_{\alpha}+\sigma_{\alpha}^{2} \right)}$.

Therefore, we obtained an upper bound on $\mu_{\alpha}$, $\mu_{\beta}$, $\sigma_{\alpha}^{2}$,$\sigma_{\beta}^{2}$, and $\sigma_{\alpha\beta}$ to achieve a reduction in cell mass variation:

$1+\frac{2\left( \mu_{\beta}+2\sigma_{\alpha\beta} \right){+\left( \mu_{\beta}+2\sigma_{\alpha\beta} \right)}^{2}+\sigma_{\beta}^{2}}{\sigma_{m}^{2}+1}\leq4e^{-2\left( \mu_{\alpha}+\sigma_{\alpha}^{2} \right)}$.

Similarly, the constraint of $\langle m(1)\rangle=2\langle m_{0}\rangle=2$ leads to a lower bound:

$2\leq e^{\mu_{\alpha}+\frac{1}{2}\sigma_{\alpha}^{2}}+\langle\beta e^{\sigma_{\alpha\beta}\frac{\beta-\mu_{\beta}}{\sigma_{\beta}^{2}}}\rangle e^{\mu_{\alpha}+\frac{1}{2}\sigma_{\alpha}^{2}\left( 1-\rho^{2} \right)}=\left( {1+\mu}_{\beta}+\sigma_{\alpha\beta} \right)e^{\mu_{\alpha}+\frac{1}{2}\sigma_{\alpha}^{2}}$.

Together, the parameter regions required to achieve cell mass CV reduction and double the average cell mass are as follows:

$1+\frac{2\left( \mu_{\beta}+2\sigma_{\alpha\beta} \right){+\left( \mu_{\beta}+2\sigma_{\alpha\beta} \right)}^{2}+\sigma_{\beta}^{2}}{\sigma_{m}^{2}+1}\leq4e^{-2\left( \mu_{\alpha}+\sigma_{\alpha}^{2} \right)}$,

$\left( {1+\mu}_{\beta}+\sigma_{\alpha\beta} \right){\geq2e}^{-\mu_{\alpha}-\frac{1}{2}\sigma_{\alpha}^{2}}$.

Combining these two inequalities, we have:

$\frac{\left( \sigma_{m}^{2}+1 \right)\left( {1+\mu}_{\beta}+\sigma_{\alpha\beta} \right)^{2}}{\left( \sigma_{m}^{2}{+\sigma}_{\beta}^{2} \right)+\left( 1+\mu_{\beta}+2\sigma_{\alpha\beta} \right)^{2}}\geq e^{\sigma_{\alpha}^{2}}$.

For simplicity, we assume $\sigma_{\alpha\beta}=0$, then:

$\left[ \sigma_{m}^{2}-\left( e^{\sigma_{\alpha}^{2}}-1 \right) \right]\left( {1+\mu}_{\beta} \right)^{2}\geq e^{\sigma_{\alpha}^{2}}\left( \sigma_{m}^{2}+\sigma_{\beta}^{2} \right)$.

This leads to the conditions:

$\mu_{\beta}\geq e^{{\frac{1}{2}\sigma}_{\alpha}^{2}}\sqrt{1+\sigma_{\beta}^{2}/\sigma_{m}^{2}}-1$ and $\sigma_{\alpha}^{2}\leq log\left( 1+\sigma_{m}^{2} \right)$.

In conclusion, in order to maintain or reduce cell mass variation throughout the cell cycle, two conditions must be met: the mean of $\beta$ should not be too small, and the variation of $\alpha$ should not be too large.

**3.1.2 Numerical simulations.**

We also validated the conclusions drawn from the analytical solutions through numerical simulations.

For universality, we normalized the correlation between growth rate and cell mass using the means of birth mass and cell cycle length as follows:

$\frac{dm^{'}}{dt^{'}}=\alpha^{'}m^{'}+\beta^{'}$,

where $m^{'}=\frac{m}{<m_{0}>}, t^{'}= \frac{t}{<T>}, \alpha^{'}=\alpha<T>,\beta^{'}=\beta\frac{<T>}{<m_{0}>}$.

For simplicity, we assumed that $\frac{\sigma_{\beta}}{<\beta>}=\frac{\sigma_{\alpha}}{<\alpha>}, \sigma_{\alpha\beta}=0$, and that cells could maintain the same division mass. Thus from Eq. 3, we have:

$2=<e^{\alpha^{'}}+\frac{\beta^{'}}{\alpha^{'}}(e^{\alpha^{'}}-1)>$.

From this, we derive:

$<\beta^{'}>=\frac{2-e^{<\alpha^{'}>+\frac{1}{2}Var(\alpha^{'})}}{e^{<\alpha^{'}>+\frac{1}{2}Var(\alpha^{'})}-1}{<\alpha}^{'}>$. (Eq. 4)

As a result, the CV of$m(t)$ only depends on the mean and variation of $\alpha^{'}$. In our simulations, we varied the mean of $\alpha^{'}$ within the range of 0 to ln(2). Previous studies in the literature have reported that the CV of cell mass growth rate typically falls between 10% and 25%(2–6). Therefore we considered a range of CV for $\alpha^{'}$ from 0 to 40%. Since all the cell lines we investigated demonstrated similar variability in birth mass (Fig 1E), we adopted a constant birth mass CV, $\sigma_{m_{0}}=0.22$. Thus the birth mass, $m_{i}^{'}\left( 0 \right),$ follows a normal distribution:

$m_{i}^{'}\left( 0 \right)\sim N\left( 1,\left( \sigma_{m_{0}} \right)^{2} \right)$.

For cell $i$, we have:

$m_{i}\left( t^{'}+\Delta t^{'} \right)=m_{i}\left( t^{'} \right)+\left( \alpha_{i}^{'}m_{i}^{'}(t^{'} \right)+{\beta_{i}}^{'})\Delta t^{'}$,

with both $\alpha_{i}^{'}$ and $\beta_{i}^{'}$ follow normal distributions:

$\alpha_{i}^{'}\sim N\left( \mu_{\alpha^{'}},\left( \mu_{\alpha^{'}}{CV}_{\alpha^{'}} \right)^{2} \right)$,

$\beta_{i}^{'}\sim N\left( \mu_{\beta^{'}},\left( \mu_{\beta^{'}}{CV}_{\alpha^{'}} \right)^{2} \right)$,

where $\mu_{\beta^{'}}$ is determined by Eq. 4.

The simulation results were plotted in S10A-B Fig and Fig 4G, confirming the findings derived from the analytical solutions:

1. The cell mass CV decreases at the beginning of the cell cycle, with the rate of reduction inversely related to the mean of $\alpha^{'}$ and unaffected by the CV of $\alpha^{'}$ (S10A Fig).
2. The rate of mass CV reduction diminishes as cell cycle progresses and can potentially turn positive towards the latter stages of the cell cycle (S10B Fig).
3. The overall change in cell mass CV over the entire cell cycle is contingent on both the mean and CV of $\alpha^{'}$.
4. Smaller mean and CV for $\alpha^{'}$ correspond to more significant reductions in cell mass CV (Fig 4G).

We further examined scenarios where growth rate variation was introduced solely to $\alpha^{'}$ or $\beta^{'}.$ Importantly, these variations did not significantly impact our major conclusions (S10C-H Fig).

**3.2 Bilinear growth rate modulation**

When growth rate modulation follows a bilinear (BI) form, the correlation between cell mass and growth rate can be expressed as

$\frac{dm}{dt}=\alpha m\left( m<m_{\tau} \right)+(\gamma m+\alpha m_{\tau}-\gamma m_{\tau})(m\geq m_{\tau})$.

We normalized this expression by the means of birth mass and cell cycle length:

$\frac{dm^{'}}{dt^{'}}=\alpha^{'}m^{'}\left( m^{'}<m_{\tau}^{'} \right)+\left( \gamma^{'}m^{'}+\alpha^{'}m_{\tau}^{'}-\gamma^{'}m_{\tau}^{'} \right)\left( m^{'}\geq m_{\tau}^{'} \right)$,

where $m^{'}=\frac{m}{<m_{0}>}, t^{'}= \frac{t}{<T>}, {\alpha^{'}=\alpha<T>, <\alpha}^{'}>=ln2,\gamma^{'}=\gamma<T>, {and m}_{\tau}^{'}=\frac{m_{\tau}}{<m_{0}>}$.

Due to the complexity of the expression above, we turned to numerical simulations to explore the change in cell mass CV throughout the cell cycle. For each cell $i$, we have:

$m_{i}\left( t^{'}+\Delta t^{'} \right)=m_{i}\left( t^{'} \right)+{[\alpha_{i}}^{'}{m_{i}}^{'}\left( {m_{i}}^{'}<m_{\tau i}^{'} \right)+\left( {\gamma_{i}}^{'}{m_{i}}^{'}+{\alpha_{i}}^{'}m_{\tau i}^{'}-{\gamma_{i}}^{'}m_{\tau i}^{'} \right)\left( {m_{i}}^{'}\geq m_{\tau i}^{'} \right)]t^{'}$.

For simplicity, we made the assumption that $\alpha'$, $\gamma'$, and $m_{\tau}'$ are independent Gaussian variables. We investigated the impact of the CV in each of these variables and compared the results to the scenario where all three variables share an equal CV (S12 A-D Fig). We found that the primary factor driving an increase in the cell mass CV is the CV associated with $\alpha^{'}$, which represents the exponential portion of the mass vs. growth correlation. Conversely, the impact of the CV in $\gamma'$ and $m_{\tau}'$ is relatively minor. Moreover, we examined the impact of the means of $\gamma'$ and $m_{\tau}'$ on the change in cell mass CV. We found that a smaller mean value of $\gamma$’, indicating a more pronounced growth rate modulation, and a smaller mean value of $m_{\tau}'$, signifying more cells being affected by the growth rate modulation, resulting in a more significant reduction in the cell mass CV (Fig 4H-I and S12E-H Fig).

1. Simulations of the contribution of each control mechanism on cell mass variation in untreated HeLa and RPE-1 cells, as well as RPE-1 cells treated with palbociclib or rapamycin.

We adopted parameters derived from experimental data to simulate the contribution of each control mechanism to cell mass variation. The meanings and values of these parameters are summarized in the Table in this section.

The CV of the cell cycle length, $CV_{T}$, was estimated from the average CV within each bin of the cell mass vs. cell cycle length correlation. For simplification, we only considered the intercellular noise of growth rate fluctuation (S11C Fig). We utilized the average slope and intercept of the cell mass vs. growth rate correlations for both the G1 and nonG1 phases to simulate mass-dependent growth rate regulation across the entire cell cycle.

At the initiation of each simulation run, we generated the division mass distribution for the cell population, which followed a normal distribution, ${md}_{i, i=1,\ldots,evt}\sim N(\mu_{md},{(\mu_{md}*CV_{md})}^{2})$. Subsequently, we conducted simulations to track the change in cell mass variation over the course of the cell cycle, culminating in the division of the subsequent generation. These simulations encompassed various scenarios designed to explore different conditions and factors influencing cell mass variation.

In the simulations, we implemented a cutoff for the entire cell cycle length, $T_{max}$. If the cell cycle length of a cell exceeds $T_{max}$, its cell cycle length is truncated at $T_{max}$.

Scenario I. Without noise or control mechanisms

The birth mass of a cell $i$ is determined by half of the mother cell division mass,

$m_{i}^{j=1}={md}_{i}/2$.

The growth rate is represented as

$gr=ln2/T$,

where $T$ is the average cell cycle length.

Thus cell mass at any given point of the cell cycle can be calculated as

$m_{i}^{j+1}=m_{i}^{j}+m_{i}^{j}gr\cdot dt$,

where $j=1,\ldots,\frac{T}{dt}-1$ is the simulation step.

Scenario II. With partition error, without control mechanisms

We used $\delta_{p}$ to represent the noise in cell partition, which follows a normal distribution, $\delta_{p}\sim N(0,{DA_{std}}^{2})$.

As a result,

$$m_{i}^{j=1}={md}_{i}\left( 0.5+\delta_{p} \right).$$

Similarly to Scenario I,

$gr=ln2/T$,

$m_{i}^{j+1}=m_{i}^{j}+m_{i}^{j}gr\cdot dt$,

$$j=1,\ldots,\frac{T}{dt}-1.$$

Scenario III. With cell cycle variation, without control mechanisms

$m_{i}^{j=1}={md}_{i}/2$,

$gr=ln2/T$.

We add Gaussian noise to the G1 and nonG1 lengths, with the noise CV equal to $CV_{T}$,

${G1}_{i}=G1\left( 1+\delta_{G1} \right),\delta_{G1}\sim N(0,{CV}_{T}^{2})$,

${nonG1}_{i}=nonG1\left( 1+\delta_{nonG1} \right)$, $\delta_{nonG1}\sim N(0,{CV}_{T}^{2})$.

The whole cell cycle length of cell $i$ is the sum of the G1 and nonG1 lengths:

$T_{i}={G1}_{i}+{nonG1}_{i}$.

$m_{i}^{j+1}=m_{i}^{j}+m_{i}^{j}gr\cdot dt$,

$$j=1,\ldots,\left\lceil\frac{T_{i}}{dt} \right\rceil-1.$$

Scenario IV. With growth rate variation, without control mechanisms

$m_{i}^{1}={md}_{i}/2$.

We introduce Gaussian noise to growth rate, with the noise CV equal to $CV_{gr}$. Then the growth rate of cell $i$ is determined by

$gr_{i}=ln2/T(1+\delta_{gr}),\delta_{gr}\sim N(0,{CV}_{gr}^{2})$.

$m_{i}^{j+1}=m_{i}^{j}+m_{i}^{j}gr_{i}\cdot dt$,

$$j=1,\ldots,\frac{T}{dt}-1.$$

Scenario V. With all noise, without control mechanisms

As described in Scenario II-IV, we incorporate Gaussian noise in cell partition, cell cycle length, and cell growth rate. For cell $i$, we have

$m_{i}^{j=1}={md}_{i}\left( 0.5+\delta_{p} \right), \delta_{p}\sim N(0,{DA_{std}}^{2})$,

$$gr_{i}=ln2/T(1+\delta_{gr}),\delta_{gr}\sim N(0,{CV}_{gr}^{2}),$$

${G1}_{i}=G1\left( 1+\delta_{G1} \right),\delta_{G1}\sim N(0,{CV}_{T}^{2})$,

${nonG1}_{i}=nonG1\left( 1+\delta_{nonG1} \right)$, $\delta_{nonG1}\sim N(0,{CV}_{T}^{2})$,

$T_{i}={G1}_{i}+{nonG1}_{i}$.

Thus cell mass at any given point of the cell cycle is determined by

$m_{i}^{j+1}=m_{i}^{j}+m_{i}^{j}gr_{i}\cdot dt$,

$$j=1,\ldots,\left\lceil\frac{T_{i}}{dt} \right\rceil-1.$$

Scenario VI. With all noise and G1 length control

Similarly to Scenario V,

$$m_{i}^{j=1}={md}_{i}\left( 0.5+\delta_{p} \right), \delta_{p}\sim N(0,{DA_{std}}^{2}),$$

$$gr_{i}=\frac{ln2}{T\left( 1+\delta_{gr} \right)},\delta_{gr}\sim N\left( 0,{CV}_{gr}^{2} \right).$$

The G1 length is determined by a bilinear function:

${G1}_{i}=[\left( m_{i}^{j=1}\leq m_{G1} \right)+\left( a_{G1}m_{G1}+b_{G1} \right)\left( m_{i}^{j=1}>m_{G1} \right)](1+\delta_{G1})$,

where $a_{G1}$, $b_{G1}$, and $m_{G1}$ are parameters of the bilinear function, and $\delta_{G1}$ is the noise term, $\delta_{G1}\sim N(0,{CV}_{T}^{2})$.

The nonG1 length follows a normal distribution with a mean of $nonG1$ and a CV of $CV_{T}$,

${nonG1}_{i}=nonG1\left( 1+\delta_{nonG1} \right)$, $\delta_{nonG1}\sim N(0,{CV}_{T}^{2})$.

The cell cycle length is the sum of the G1 and nonG1 lengths,

$T_{i}={G1}_{i}+{nonG1}_{i}$.

$m_{i}^{j+1}=m_{i}^{j}+m_{i}^{j}gr_{i}\cdot dt$,

$$j=1,\ldots,\left\lceil\frac{T_{i}}{dt} \right\rceil-1.$$

Scenario VII. with all noise and nonG1 length control

Similarly to Scenario V,

$$m_{i}^{j=1}={md}_{i}\left( 0.5+\delta_{p} \right), \delta_{p}\sim N(0,{DA_{std}}^{2}),$$

$$gr_{i}=ln2/T(1+\delta_{gr}),\delta_{gr}\sim N(0,{gr_{CV}}^{2}).$$

The G1 length follows a normal distribution with a mean of $G1$ and a CV of $CV_{T}$,

${G1}_{i}=G1\left( 1+\delta_{G1} \right)$, $\delta_{G1}\sim N(0,{{CV}_{T}}^{2})$.

Thus the cell mass at any given point of the G1 phase is determined by

$m_{i}^{j_{1}+1}=m_{i}^{j_{1}}+m_{i}^{j_{1}}gr_{i}\cdot dt$,

$$j_{1}=1,\ldots,\left\lceil\frac{{G1}_{i}}{dt} \right\rceil-1.$$

The cell mass at the G1/S transition is

$m_{i}^{G1\_S}=m_{i}^{\left\lceil\frac{{G1}_{i}}{dt} \right\rceil}$.

The nonG1 length is determined by a bilinear function,

${nonG1}_{i}=[\left( m_{i}^{G1\_S}\leq m_{nonG1} \right)+\left( a_{nonG1}m_{nonG1}+b_{nonG1} \right)\left( m_{i}^{G1\_S}>m_{nonG1} \right)](1+\delta_{nonG1})$,

where $a_{nonG1}$, $b_{nonG1}$, and $m_{nonG1}$ are parameters of the bilinear function, and $\delta_{nonG1}$ is the noise term, $\delta_{nonG1}\sim N(0,{CV}_{T}^{2})$.

The cell mass during the nonG1 phase is determined by

$m_{i}^{\left\lceil\frac{{G1}_{i}}{dt} \right\rceil+j_{2}+1}=m_{i}^{\left\lceil\frac{{G1}_{i}}{dt} \right\rceil+j_{2}}+m_{i}^{\left\lceil\frac{{G1}_{i}}{dt} \right\rceil+j_{2}}gr_{i}\cdot dt$,

$$j_{2}=1,\ldots,\left\lceil\frac{{nonG1}_{i}}{dt} \right\rceil-1.$$

Scenario VIII. With all noise and growth rate control

Similarly to Scenario V, we incorporate Gaussian noise in cell partition and cell cycle length:

$m_{i}^{j=1}={md}_{i}\left( 0.5+\delta_{p} \right), \delta_{p}\sim N(0,{DA_{std}}^{2})$,

${G1}_{i}=G1\left( 1+\delta_{G1} \right),\delta_{G1}\sim N(0,{{CV}_{T}}^{2})$,

${nonG1}_{i}=nonG1\left( 1+\delta_{nonG1} \right)$, $\delta_{nonG1}\sim N(0,{{CV}_{T}}^{2})$,

$$T_{i}={G1}_{i}+{nonG1}_{i}.$$

The cell growth rate is determined by a subexponential correlation between cell mass and growth rate, and we introduce Gaussian noise to both the slope and the intercept terms of the correlation:

$m_{i}^{j+1}=m_{i}^{j}+{(m}_{i}^{j}\alpha_{i}+\beta_{i})\cdot dt$,

with $\alpha_{i}=\alpha\left( 1+\delta_{gr} \right),\delta_{gr}\sim N\left( 0,{CV}_{gr}^{2} \right),$

and $\beta_{i}=\beta\left( 1+\delta_{gr} \right),\delta_{gr}\sim N\left( 0,{CV}_{gr}^{2} \right).$

$$j=1,\ldots,\left\lceil\frac{T_{i}}{dt} \right\rceil-1.$$

Scenario IX. With all noise and all control mechanisms

As described in previous scenarios, we introduce Gaussian noise to cell partition:

$$m_{i}^{j=1}={md}_{i}\left( 0.5+\delta_{p} \right), \delta_{p}\sim N\left( 0,{DA_{std}}^{2} \right).$$

The G1 length is determined by a bilinear function,

${G1}_{i}=[\left( m_{i}^{1}\leq m_{G1} \right)+\left( a_{G1}m_{G1}+b_{G1} \right)\left( m_{i}^{1}>m_{G1} \right)](1+\delta_{G1})$,

where $a_{G1}$, $b_{G1}$, and $m_{G1}$ are parameters of the bilinear function, and $\delta_{G1}$ is the noise term, $\delta_{G1}\sim N(0,{CV}_{T}^{2})$.

Similarly, the nonG1 length is determined by another bilinear function,

${nonG1}_{i}=[\left( m_{i}^{G1\_S}\leq m_{nonG1} \right)+\left( a_{nonG1}m_{nonG1}+b_{nonG1} \right)\left( m_{i}^{G1\_S}>m_{nonG1} \right)](1+\delta_{nonG1})$, $\delta_{nonG1}\sim N(0,{{CV}_{T}}^{2})$,

where $a_{nonG1}$, $b_{nonG1}$, and $m_{nonG1}$ are parameters of the bilinear function; $m_{i}^{G1\_S}$ is the cell mass at the G1/S transition, $m_{i}^{G1\_S}=m_{i}^{\left\lceil\frac{{G1}_{i}}{dt} \right\rceil}$; and $\delta_{nonG1}$ is the noise term, $\delta_{nonG1}\sim N(0,{CV}_{T}^{2})$.

The cell growth rate is determined by a subexponential correlation between cell mass and growth rate, and we introduce Gaussian noise to both the slope and the intercept terms of the correlation:

$$\alpha_{i}=\alpha\left( 1+\delta_{gr} \right),\delta_{gr}\sim N\left( 0,{CV}_{gr}^{2} \right),$$

$\beta_{i}=\beta\left( 1+\delta_{gr} \right),\delta_{gr}\sim N\left( 0,{CV}_{gr}^{2} \right).$

Thus, the cell mass during the G1 phase is determined by

$m_{i}^{j_{1}+1}=m_{i}^{j_{1}}+{(m}_{i}^{j_{1}}\alpha_{i}+\beta_{i})\cdot dt$,

$$j_{1}=1,\ldots,\left\lceil\frac{{G1}_{i}}{dt} \right\rceil-1;$$

whereas the cell mass during the nonG1 phase is determined by

$m_{i}^{\left\lceil\frac{{G1}_{i}}{dt} \right\rceil+j2+1}=m_{i}^{\left\lceil\frac{{G1}_{i}}{dt} \right\rceil+j2}+{(m}_{i}^{\left\lceil\frac{{G1}_{i}}{dt} \right\rceil+j2}\alpha_{i}+\beta_{i})\cdot dt$,

$$j_{2}=1,\ldots,\left\lceil\frac{{nonG1}_{i}}{dt} \right\rceil-1.$$

The simulated division mass CVs for each scenario under different treatment conditions were summarized in S8 Table.

Table. Parameters used in the simulations in this section.

|  | meaning | HeLa | RPE | RPE Palb | RPE Rapa |
| --- | --- | --- | --- | --- | --- |
| $evt$ | Number of cells | 1000 | 1000 | 1000 | 1000 |
| $T$ | Average cell cycle length | 28.0 | 20.0 | 24.2 | 33.5 |
| $G1$ | Average G1 length | 11.1 | 7.8 | 12.3 | 17.4 |
| $nonG1$ | Average nonG1 length | 17.1 | 12.3 | 11.9 | 16.1 |
| $dt$ | Simulation step size | 0.5 | 0.5 | 0.5 | 0.5 |
| $CV_{T}$ | CV of cell cycle phase length | 0.25 | 0.25 | 0.25 | 0.25 |
| $T_{max}$ | Maximum cell cycle length allowed in the simulation | 48 | 48 | 48 | 72 |
| $\mu_{md}$ | Average division mass | 897 | 785 | 1139 | 556 |
| $CV_{md}$ | Division mass CV at the initiation of the simulation | 0.18 | 0.23 | 0.21 | 0.20 |
| $D.A._{std}$ | Standard deviation of Division Asymmetry | 0.053 | 0.051 | 0.055 | 0.050 |
| $CV_{gr}$ | CV of growth rate | 0.23 | 0.33 | 0.43 | 0.30 |
| $a_{G1}$ | Slope of the birth mass vs. G1 length correlation | -0.0165 | -0.0132 | -0.0067 | -0.0889 |
| $b_{G1}$ | Intercept of the birth mass vs. G1 length correlation | 19.2 | 12.2 | 16.0 | 40.9 |
| $m_{G1}$ | Transition mass of the birth mass vs. G1 length correlation | 595 | 483 | Inf | 334 |
| $a_{nonG1}$ | Slope of the G1/S mass vs. nonG1 length correlation | -0.0237 | -0.0152 | -0.0294 | -0.0141 |
| $b_{nonG1}$ | Intercept of the G1/S mass vs. nonG1 length correlation | 30.9 | 18.6 | 31.95 | 21.2 |
| $m_{nonG1}$ | Transition of the G1/S mass vs. nonG1 length correlation | 699 | 459 | 701 | 387 |
| $\alpha$ | Slope of the cell mass vs. growth rate correlation | 0.0177 | 0.0141 | -0.00007 | 0.0158 |
| $\beta$ | Intercept of the cell mass vs. growth rate correlation | 6.87 | 9.74 | 22.20 | 1.47 |

**References**

1. Thomas P, Terradot G, Danos V, Weiße AY. Sources, propagation and consequences of stochasticity in cellular growth. Nat Commun [Internet]. 2018;9(1):1–11. Available from: http://dx.doi.org/10.1038/s41467-018-06912-9

2. Son S, Tzur A, Weng Y, Jorgensen P, Kim J, Kirschner MW, et al. Direct observation of mammalian cell growth and size regulation. Nat Methods [Internet]. 2012 Sep [cited 2012 Nov 5];9(9):910–2. Available from: http://www.ncbi.nlm.nih.gov/pubmed/22863882

3. Mu L, Kang JH, Olcum S, Payer KR, Calistri NL, Kimmerling RJ, et al. Mass measurements during lymphocytic leukemia cell polyploidization decouple cell cycle- And cell size-dependent growth. Proc Natl Acad Sci U S A [Internet]. 2020 Jul 7;117(27):15659–65. Available from: http://biorxiv.org/cgi/content/short/2019.12.17.879080v1?rss=1&utm_source=researcher_app&utm_medium=referral&utm_campaign=RESR_MRKT_Researcher_inbound

4. Godin M, Delgado FF, Son S, Grover WH, Bryan AK, Tzur A, et al. Using buoyant mass to measure the growth of single cells. Nat Methods [Internet]. 2010 [cited 2013 Jan 10];7(5):387–90. Available from: http://www.nature.com/nmeth/journal/vaop/ncurrent/full/nmeth.1452.html

5. Miettinen TP, Ly KS, Lam A, Manalis SR. Single-cell monitoring of dry mass and dry mass density reveals exocytosis of cellular dry contents in mitosis. Elife [Internet]. 2022 May 10;11:1–20. Available from: https://elifesciences.org/articles/76664

6. Mir M, Wang Z, Shen Z, Bednarz M, Bashir R, Golding I, et al. Optical measurement of cycle-dependent cell growth. Proc Natl Acad Sci U S A [Internet]. 2011 Aug 9 [cited 2013 May 22];108(32):13124–9. Available from: http://www.pubmedcentral.nih.gov/articlerender.fcgi?artid=3156192&tool=pmcentrez&rendertype=abstract
