# Supplementary material for: Cell size homeostasis is tightly controlled throughout the cell cycle
Source: PLoS Biol. 2024 Jan 5;22(1):e3002453. doi: 10.1371/journal.pbio.3002453 (PMC10769027; doi:10.1371/journal.pbio.3002453)
Supplement: S1 Table — (DOCX) [file pbio.3002453.s015.docx]

**Table S1. Characteristics of the human cell lines used in this study.**

|  | RPE-1 | HeLa | U2OS | Saos-2 | HT1080 |
| --- | --- | --- | --- | --- | --- |
| morphology | epithelial | epithelial | epithelial | epithelial | epithelial |
| tissue | Eye; Pigmented epithelium; Retina | Uterus; Cervix | Bone | Bone | Connective tissue |
| disease | Normal | Adenocarcinoma | Osteosarcoma | Osteosarcoma | Fibrosarcoma |
| gender and age | Female, 1 | Female, 31 | Female, 15 | Female, 11 | Male, 35 |
| Karyotype | Modal chromosome number of 46, near-diploid | Modal number = 82, 1.76 fold of RPE-1* | 1.37 fold of RPE-1* | 1.33 fold of RPE-1* | 2.01 fold of RPE-1* |
| G1/S circuitry | Intact | inactivated pRb, p103, p170, p21, p27, p53 [1] | deficient p16, p53, Wip1; intact pRb[2-5] | deleted pRb and p53[2,3,5] | deleted p16; mutated p53 and N-ras; intact pRB[5-7] |

| * measured by this study using Hoechst stain. |
| --- |
| [1]Moody, Cary A., and Laimonis A. Laimins. "Human papillomavirus oncoproteins: pathways to transformation." Nature Reviews Cancer 10.8 (2010): 550-560. |
| [2]Diller, Lisa, et al. "p53 functions as a cell cycle control protein in osteosarcomas." Molecular and cellular biology 10.11 (1990): 5772-5781. |
| [3]Stott, Francesca J., et al. "The alternative product from the human CDKN2A locus, p14ARF, participates in a regulatory feedback loop with p53 and MDM2." The EMBO journal 17.17 (1998): 5001-5014. |
| [4]Kleiblova, Petra, et al. "Gain-of-function mutations of PPM1D/Wip1 impair the p53-dependent G1 checkpoint." Journal of Cell Biology 201.4 (2013): 511-521. |
| [5]Moolmuang, Benchamart, and Michael A. Tainsky. "CREG1 enhances p16INK4a-induced cellular senescence." *Cell Cycle* 10.3 (2011): 518-530. |
| [6]Anderson, Michael J., et al. "Evidence that wild‐type TP53, and not genes on either chromosome 1 or 11, controls the tumorigenic phenotype of the human fibrosarcoma HT1080." Genes, Chromosomes and Cancer 9.4 (1994): 266-281. |
| [7]Brown, Robin, et al. "Mechanism of activation of an N‐ras gene in the human fibrosarcoma cell line HT1080." The EMBO journal 3.6 (1984): 1321-1326. |
