# Supplementary material for: Cell size homeostasis is tightly controlled throughout the cell cycle
Source: PLoS Biol. 2024 Jan 5;22(1):e3002453. doi: 10.1371/journal.pbio.3002453 (PMC10769027; doi:10.1371/journal.pbio.3002453)
Supplement: S2 Table — MAD is the median absolute deviation, and nMAD is MAD normalized by the median in robust statistics. (DOCX) [file pbio.3002453.s016.docx]

**Table S2. The durations of cell cycle phases for HeLa, RPE-1, RPE-1 in 100 nM rapamycin or 50 nM palbociclib at cell mass homeostasis.** MAD is the median absolute deviation, and nMAD is MAD normalized by the median in robust statistics.

|  |  | mean (hour) | median (hour) | std (hour) | MAD (hour) | CV | nMAD |
| --- | --- | --- | --- | --- | --- | --- | --- |
| HeLa | Cell cycle | 28.0 | 27.5 | 4.6 | 3.6 | 0.16 | 0.13 |
|  | G1 | 11.1 | 10.5 | 4.3 | 3.2 | 0.38 | 0.30 |
|  | S | 11.3 | 11.0 | 4.2 | 3.1 | 0.37 | 0.28 |
|  | G2-M | 5.8 | 5.5 | 2.7 | 2.0 | 0.46 | 0.36 |
| RPE-1 | Cell cycle | 18.0 | 18.0 | 3.1 | 2.3 | 0.17 | 0.13 |
|  | G1 | 7.8 | 7.5 | 3.2 | 2.4 | 0.41 | 0.32 |
|  | S | 10.0 | 9.5 | 3.2 | 2.4 | 0.32 | 0.25 |
|  | G2-M | 3.1 | 3.0 | 3.2 | 1.7 | 0.54 | 0.39 |
| RPE-1 Rapa | Cell cycle | 32.6 | 32.0 | 8.2 | 7.0 | 0.25 | 0.22 |
|  | G1 | 16.7 | 15.5 | 9.4 | 7.3 | 0.56 | 0.47 |
|  | S | 14.1 | 13.5 | 4.4 | 3.2 | 0.31 | 0.24 |
|  | G2-M | 3.9 | 3.5 | 2.5 | 1.6 | 0.64 | 0.46 |
| RPE-1 Palb | Cell cycle | 26.0 | 25.3 | 5.3 | 4.1 | 0.20 | 0.16 |
|  | G1 | 12.3 | 11.0 | 7.0 | 5.3 | 0.57 | 0.48 |
|  | S | 11.9 | 11.0 | 6.8 | 5.0 | 0.58 | 0.45 |
|  | G2-M | 2.8 | 2.4 | 2.2 | 1.3 | 0.77 | 0.55 |
