# Supplementary material for: Cell size homeostasis is tightly controlled throughout the cell cycle
Source: PLoS Biol. 2024 Jan 5;22(1):e3002453. doi: 10.1371/journal.pbio.3002453 (PMC10769027; doi:10.1371/journal.pbio.3002453)
Supplement: S3 Table — (DOCX) [file pbio.3002453.s017.docx]

**Table S3. Comparing cell cycle phase durations and mass versus phase length correlations with and without the mTurquoise2-SLBP marker in HeLa cells.**

|  |  | HeLa mAG-hGeminin | HeLa mAG-Geminin mTurq2-SLBP |
| --- | --- | --- | --- |
| Median duration (hour) | Cell cycle | 26.0 | 27.5 |
|  | G1 | 10.5 | 10.5 |
|  | nonG1 | 16.5 | 16.5 |
| Mass-length Pearson correlation | Birth mass-Cell cycle | -0.27 | -0.26 |
|  | Birth mass-G1 | -0.19 | -0.20 |
|  | G1/S mass-nonG1 | -0.32 | -0.34 |
