# Supplementary material for: Cell size homeostasis is tightly controlled throughout the cell cycle
Source: PLoS Biol. 2024 Jan 5;22(1):e3002453. doi: 10.1371/journal.pbio.3002453 (PMC10769027; doi:10.1371/journal.pbio.3002453)
Supplement: S4 Table — The significantly better fits (p_bilinear or p_linear < 0.05) and the significant negative correlations (p < 0.05) are highlighted. (DOCX) [file pbio.3002453.s018.docx]

**Table S4. Comparison of the linear and bilinear fits for the cell mass vs. cell cycle phase length correlations.** The significantly better fits (p_bilinear or p_linear < 0.05) and the significant negative correlations (p<0.05) are highlighted.

|  |  | AICc_bilinear | AICc_linear | p_bilinear | p_linear | R | p-Value of R |
| --- | --- | --- | --- | --- | --- | --- | --- |
| HeLa | Birth mass-G1 length | -61.8 | -41.8 | 4.6E-05 |  | -0.20 | 1.4E-84 |
|  | G1/S mass-S length | -65.4 | -14.4 | 8.3E-12 |  | -0.29 | 3.1E-141 |
|  | S/G2 mass-G2-M length | -103.1 | -79.4 | 7.1E-06 |  | -0.18 | 8.5E-66 |
|  | Birth mass-cell cycle length | -22.8 | -6.1 | 2.4E-04 |  | -0.26 | 5.8E-61 |
| RPE-1 | Birth mass-G1 length | -45.0 | -39.9 | 0.07 |  | -0.25 | 3.6E-55 |
|  | G1/S mass-S length | -43.4 | -22.7 | 3.2E-05 |  | -0.12 | 3.5E-12 |
|  | S/G2 mass-G2-M length | -91.9 | -92.9 |  | 0.58 | -0.08 | 2.3E-13 |
|  | Birth mass-cell cycle length | -15.2 | -8.0 | 0.03 |  | -0.25 | 1.6E-12 |
