# Supplementary material for: Cell size homeostasis is tightly controlled throughout the cell cycle
Source: PLoS Biol. 2024 Jan 5;22(1):e3002453. doi: 10.1371/journal.pbio.3002453 (PMC10769027; doi:10.1371/journal.pbio.3002453)
Supplement: S5 Table — The significantly better fits (p_bilinear or p_linear < 0.05) are highlighted. (DOCX) [file pbio.3002453.s019.docx]

**Table S5. Comparison of the linear and bilinear fits for the cell mass vs. growth rate correlations.** The significantly better fits (p_bilinear or p_linear < 0.05) are highlighted.

|  |  | AICc_bilinear | AICc_linear | p_bilinear | p_linear |
| --- | --- | --- | --- | --- | --- |
| HeLa | All | -41.2 | -30.0 | 0.003 |  |
|  | G1 | -12.3 | -17.2 |  | 0.09 |
|  | nonG1 | -13.0 | -14.0 |  | 0.61 |
|  | early G1 | -2.5 | -5.5 |  | 0.22 |
|  | late G1 | -5.4 | -11.1 |  | 0.06 |
|  | S | -19.8 | -27.7 |  | 0.02 |
|  | G2-M | 3.0 | -3.4 |  | 0.04 |
|  | Stage 0.85/1 | 13.7 | 16.0 | 0.32 |  |
| RPE-1 | G1 | -3.1 | -11.4 |  | 0.02 |
|  | nonG1 | -15.9 | -25.0 |  | 0.01 |
| U2OS | G1 | 21.0 | 25.9 | 0.09 |  |
|  | nonG1 | -6.4 | -11.1 |  | 0.09 |
| HT1080 | G1 | 9.7 | 13.2 | 0.18 |  |
|  | nonG1 | -0.7 | 9.4 | 0.007 |  |
| Saos-2 | G1 | -28.5 | -31.0 |  | 0.29 |
|  | nonG1 | -41.5 | -35.7 | 0.05 |  |
