# Supplementary material for: Cell size homeostasis is tightly controlled throughout the cell cycle
Source: PLoS Biol. 2024 Jan 5;22(1):e3002453. doi: 10.1371/journal.pbio.3002453 (PMC10769027; doi:10.1371/journal.pbio.3002453)
Supplement: S6 Table — For correlations fitted better by the linear model, dmdt=αm+β, the normalized parameters α′ and β′ are listed in the table, with α′ = α, β′=β, where and are the means of cell cycle length and cell birth mass, respectively. For exponential growth, α′ = ln2 ~= 0.693. For correlations fitted better by the bilinear model, dmdt=(am+b)(m<mτ)+(γm+amτ+b-γmτ)(m≥mτ), the normalized parameters a′, b′, γ′, and mτ′ are listed in the table, with a′ = a, b′=b,γ′=γ,mτ′=mτ. The correlation slopes, α′, a′, and γ′, lower than 0.75 or higher than 1.25-fold (arbitrarily chosen thresholds) of ln2 were highlighted. SE and BI denote the type of growth rate modulation, where SE stands for sub-exponential and BI stands for bilinear. (DOCX) [file pbio.3002453.s020.docx]

**Table S6. The normalized fitting parameters for the cell mass vs. growth rate correlations for different cell lines.** For correlations fitted better by the linear model, $\frac{dm}{dt}=\alpha m+\beta$, the normalized parameters $\alpha^{'}$ and $\beta^{'}$ are listed in the table, with $\alpha^{'}=\alpha<T>,\beta^{'}=\beta\frac{<T>}{<m_{b}>}$, where $<T>$ and ${<m}_{b}>$ are the means of cell cycle length and cell birth mass, respectively. For exponential growth, $\alpha^{'}=ln2=0.693$. For correlations fitted better by the bilinear model, $\frac{dm}{dt}=\left( am+b \right)\left( m<m_{\tau} \right)+(\gamma m+am_{\tau}+b-\gamma m_{\tau})(m\geq m_{\tau})$, the normalized parameters $a^{'}$, $b^{'}$, $\gamma^{'}$, and $m_{\tau}^{'}$ are listed in the table, with $a^{'}=a<T>,b^{'}=b\frac{<T>}{<m_{b}>},\gamma^{'}=\gamma<T>, m_{\tau}^{'}=\frac{m_{\tau}}{<m_{b}>}$. The correlation slopes, $\alpha^{'}$, $a^{'}$, and $\gamma^{'}$, lower than 0.75 or higher than 1.25 fold (arbitrarily chosen thresholds) of $ln2$ were highlighted. SE and BI denote the type of growth rate modulation, where SE stands for Sub-exponential and BI stands for Bilinear.

|  |  | $\alpha^{'}, \beta'$ | $a^{'}, b^{'}, \gamma^{'}, m_{\tau}^{'}$ | Modulation type |
| --- | --- | --- | --- | --- |
| RPE-1 | G1 | 0.25, 0.44 |  | SE |
|  | nonG1 | 0.26, 0.57 |  | SE |
| HeLa | G1 | 0.40, 0.36 |  | SE |
|  | nonG1 | 0.43, 0.43 |  | SE |
| U2OS | G1 |  | 0.67, -0.16, -3.18, 1.69 | BI |
|  | nonG1 | 0.67, 0.15 |  | None |
| HT1080 | G1 |  | 0.85, -0.10, 0.16, 1.05 | BI |
|  | nonG1 |  | 0.54, 0.32, 0.08, 1.58 | BI |
| Saos-2 | G1 | 0.84, -0.15 |  | None |
|  | nonG1 |  | 0.96, -0.30, 0.42, 1.53 | BI |
