# Supplementary material for: Cell size homeostasis is tightly controlled throughout the cell cycle
Source: PLoS Biol. 2024 Jan 5;22(1):e3002453. doi: 10.1371/journal.pbio.3002453 (PMC10769027; doi:10.1371/journal.pbio.3002453)
Supplement: S7 Table — (DOCX) [file pbio.3002453.s021.docx]

**Table S7. The values of** $\boldsymbol{\lambda}^{\boldsymbol{'}}$ **and** $\boldsymbol{\alpha'}$ **used in Fig. 5L, for untreated HeLa and RPE-1 cells, as well as RPE-1 cells treated with 50 nM palbociclib or 100 nM rapamycin.**

|  | $\lambda^{'}$ | $\alpha'$ |
| --- | --- | --- |
| HeLa | -0.47 | 0.41 |
| RPE-1 | -0.22 | 0.25 |
| RPE-1 Palb | -0.15 | -0.002 |
| RPE-1 Rapa | -0.45 | 0.55 |
