# Supplementary material for: Cell size homeostasis is tightly controlled throughout the cell cycle
Source: PLoS Biol. 2024 Jan 5;22(1):e3002453. doi: 10.1371/journal.pbio.3002453 (PMC10769027; doi:10.1371/journal.pbio.3002453)
Supplement: S8 Table — The values reported in this table are the average of division mass CVs obtained from 50 simulations. (DOCX) [file pbio.3002453.s022.docx]

**Table S8: Contribution of each factor to cell mass variation, as indicated by the division mass CV simulated using the stochastic model (SI Text, Section 4).** The values reported in this table are the average division mass CV obtained from 50 simulations.

|  | HeLa | RPE | RPE Palb | RPE Rapa |
| --- | --- | --- | --- | --- |
| I. without noise or control mechanisms | 0.18 | 0.23 | 0.21 | 0.20 |
| II. with partition noise, without control mechanisms | 0.21 | 0.25 | 0.24 | 0.22 |
| III. with cell cycle variation, without control mechanisms | 0.22 | 0.26 | 0.24 | 0.24 |
| IV. with growth rate variation, without control mechanisms | 0.24 | 0.33 | 0.37 | 0.29 |
| V. with all noise, without control mechanisms | 0.30 | 0.38 | 0.43 | 0.35 |
| VI. with all noise and G1 length control | 0.28 | 0.35 | 0.41 | 0.28 |
| VII. with all noise and nonG1 length control | 0.25 | 0.37 | 0.40 | 0.34 |
| VIII. with all noise and growth rate control | 0.23 | 0.24 | 0.26 | 0.28 |
| IX. with all noise and all control mechanisms | 0.18 | 0.21 | 0.24 | 0.22 |
