# Supplementary material for: Cell size homeostasis is tightly controlled throughout the cell cycle
Source: PLoS Biol. 2024 Jan 5;22(1):e3002453. doi: 10.1371/journal.pbio.3002453 (PMC10769027; doi:10.1371/journal.pbio.3002453)
Supplement: S9 Table — (DOCX) [file pbio.3002453.s023.docx]

**Table S9. The frequencies of cell death, cell cycle arrest, and cytoplasmic loss observed in the long–term measurements in HeLa, RPE-1, RPE-1 in 100 nM rapamycin, and RPE-1 in 50 nM palbociclib when cells have reached cell mass homeostasis.**

|  | HeLa | RPE | RPE Rapa | RPE Palb |
| --- | --- | --- | --- | --- |
| Death | ~2% | <0.1% | <0.1% | ~2% |
| Cell cycle arrest | ~0.2% | ~0.2% | <0.1% | ~0.3% |
| Cytoplasmic loss during mitosis | <0.1% | <0.1% | <0.1% | ~0.5% |
