# Supplementary material for: Cell size homeostasis is tightly controlled throughout the cell cycle
Source: PLoS Biol. 2024 Jan 5;22(1):e3002453. doi: 10.1371/journal.pbio.3002453 (PMC10769027; doi:10.1371/journal.pbio.3002453)
Supplement: S10 Table — (DOCX) [file pbio.3002453.s024.docx]

**Table S10. Birth size CVs, division size CVs, and DA stds. reported in the literature.**

|  | Birth size CV | Division size CV | DA std. |  |  |  |  |  |  |  |  |  |  |  |  |  |  |  |  |  |  |  |  |
| --- | --- | --- | --- | --- | --- | --- | --- | --- | --- | --- | --- | --- | --- | --- | --- | --- | --- | --- | --- | --- | --- | --- | --- |
| *C. crescentus*[1] | 16-18% | 12% |  |  |  |  |  |  |  |  |  |  |  |  |  |  |  |  |  |  |  |  |  |
| *E. coli* | 12%[1] | 11%[1] | 1%[3] |  |  |  |  |  |  |  |  |  |  |  |  |  |  |  |  |  |  |  |  |
| *M. smegmatis*[2] | 19% |  |  |  |  |  |  |  |  |  |  |  |  |  |  |  |  |  |  |  |  |  |  |
| *M. bovis* BCG[2] | 20% |  |  |  |  |  |  |  |  |  |  |  |  |  |  |  |  |  |  |  |  |  |  |
| *H. salinarum*[3] | 16% | 13% | 3% |  |  |  |  |  |  |  |  |  |  |  |  |  |  |  |  |  |  |  |  |
| *S. cerevisiae*[4] | 24% | 23% |  |  |  |  |  |  |  |  |  |  |  |  |  |  |  |  |  |  |  |  |  |
| *S. pombe*[5] |  | 6% | 1.6% |  |  |  |  |  |  |  |  |  |  |  |  |  |  |  |  |  |  |  |  |
| *Arabidopsis* shoot stem cell[6] | 24% | 13% |  |  |  |  |  |  |  |  |  |  |  |  |  |  |  |  |  |  |  |  |  |
| L1210 | 25%[7], 8%[9] | 7%[9] | 4.8%[10] |  |  |  |  |  |  |  |  |  |  |  |  |  |  |  |  |  |  |  |  |
| MOLT4[7] | 29% |  |  |  |  |  |  |  |  |  |  |  |  |  |  |  |  |  |  |  |  |  |  |
| RBL[8] | 11% | 11% |  |  |  |  |  |  |  |  |  |  |  |  |  |  |  |  |  |  |  |  |  |
| RAW 264.7[8] | 16% |  |  |  |  |  |  |  |  |  |  |  |  |  |  |  |  |  |  |  |  |  |  |
| FL5.12[9] | 13% | 11.50% |  |  |  |  |  |  |  |  |  |  |  |  |  |  |  |  |  |  |  |  |  |
| RKO[10] |  |  | 6.8% |  |  |  |  |  |  |  |  |  |  |  |  |  |  |  |  |  |  |  |  |
| HT-29[10] |  |  | 6.4% |  |  |  |  |  |  |  |  |  |  |  |  |  |  |  |  |  |  |  |  |
| L-929[11] | 16% | 12% | 4.8% |  |  |  |  |  |  |  |  |  |  |  |  |  |  |  |  |  |  |  |  |
|  |  |  |  |  |  |  |  |  |  |  |  |  |  |  |  |  |  |  |  |  |  |  |  |
| [1]Campos, Manuel, et al. "A constant size extension drives bacterial cell size homeostasis." Cell 159.6 (2014): 1433-1446. | | | |  |  |  |  |  |  |  |  |  |  |  |  |  |  |  |  |  |  |  |  |
| [2]Logsdon, Michelle M., et al. "A parallel adder coordinates mycobacterial cell-cycle progression and cell-size homeostasis in the context of asymmetric growth and organization." Current Biology 27.21 (2017): 3367-3374. | | | |  |  |  |  |  |  |  |  |  |  |  |  |  |  |  |  |  |  |  |  |
| [3]Eun, Ye-Jin, et al. "Archaeal cells share common size control with bacteria despite noisier growth and division." Nature microbiology 3.2 (2018): 148-154. | | | |  |  |  |  |  |  |  |  |  |  |  |  |  |  |  |  |  |  |  |  |
| [4]Barber, Felix, Ariel Amir, and Andrew W. Murray. "Cell-size regulation in budding yeast does not depend on linear accumulation of Whi5." Proceedings of the National Academy of Sciences 117.25 (2020): 14243-14250. | | | |  |  |  |  |  |  |  |  |  |  |  |  |  |  |  |  |  |  |  |  |
| [5]Sveiczer, A., B. Novak, and J. M. Mitchison. "The size control of fission yeast revisited." Journal of cell science 109.12 (1996): 2947-2957. | | | |  |  |  |  |  |  |  |  |  |  |  |  |  |  |  |  |  |  |  |  |
| [6]D’Ario, Marco, et al. "Cell size controlled in plants using DNA content as an internal scale." Science 372.6547 (2021): 1176-1181. | | | |  |  |  |  |  |  |  |  |  |  |  |  |  |  |  |  |  |  |  |  |
| [7]Tzur, Amit, et al. "Cell growth and size homeostasis in proliferating animal cells." Science 325.5937 (2009): 167-171. | | | |  |  |  |  |  |  |  |  |  |  |  |  |  |  |  |  |  |  |  |  |
| [8]Varsano, Giulia, Yuedi Wang, and Min Wu. "Probing mammalian cell size homeostasis by channel-assisted cell reshaping." Cell reports 20.2 (2017): 397-410. | | | |  |  |  |  |  |  |  |  |  |  |  |  |  |  |  |  |  |  |  |  |
| [9]Son, Sungmin, et al. "Direct observation of mammalian cell growth and size regulation." Nature methods 9.9 (2012): 910-912. | | | |  |  |  |  |  |  |  |  |  |  |  |  |  |  |  |  |  |  |  |  |
| [10]Sung, Yongjin, et al. "Size homeostasis in adherent cells studied by synthetic phase microscopy." Proceedings of the National Academy of Sciences 110.41 (2013): 16687-16692. | | | |  |  |  |  |  |  |  |  |  |  |  |  |  |  |  |  |  |  |  |  |
| [11]Killander, D., and A. Zetterberg. "Quantitative cytochemical studies on interphase growth: I. Determination of DNA, RNA and mass content of age determined mouse fibroblasts in vitro and of intercellular variation in generation time." Experimental cell research 38.2 (1965): 272-284. | | | |  |  |  |  |  |  |  |  |  |  |  |  |  |  |  |  |  |  |  |  |
