# Supplementary figures and images for: Cell size homeostasis is tightly controlled throughout the cell cycle
Source: PLoS Biol. 2024 Jan 5;22(1):e3002453. doi: 10.1371/journal.pbio.3002453 (PMC10769027; doi:10.1371/journal.pbio.3002453)

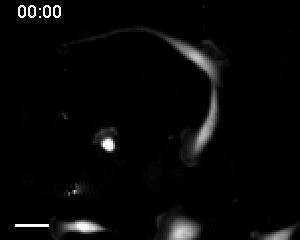

Supplement: S1 Movie — (GIF) [file pbio.3002453.s025.gif]
